# The homing of exogenous hair follicle mesenchymal stem cells into hair follicle niches

**DOI:** 10.1172/jci.insight.173549

**Published:** 2023-12-22

**Authors:** Kaitao Li, Fang Liu, Ye He, Qian Qu, Pingping Sun, Lijuan Du, Jin Wang, Ruosi Chen, Yuyang Gan, Danlan Fu, Zhexiang Fan, Bingcheng Liu, Zhiqi Hu, Yong Miao

**Affiliations:** 1 Department of Plastic and Aesthetic Surgery, Nanfang Hospital of Southern Medical University, Guangzhou, China.; 2 Medical Cosmetic and Plastic Surgery Center, The Sixth Affiliated Hospital, Sun Yat-sen University, Guangzhou, China.

**Keywords:** Dermatology, Stem cells, Chemokines, Skin, Stem cell transplantation

## Abstract

Hair loss is a debilitating condition associated with the depletion of dermal papilla cells (DPCs), which can be replenished by dermal sheath cells (DSCs). Hence, strategies aimed at increasing the populations of DPCs and DSCs hold promise for the treatment of hair loss. In this study, we demonstrated in mice that introduced exogenous DPCs and DSCs (hair follicle mesenchymal stem cells) could effectively migrate and integrate into the dermal papilla and dermal sheath niches, leading to enhanced hair growth and prolonged anagen phases. However, the homing rates of DPCs and DSCs were influenced by various factors, including recipient mouse depilation, cell passage number, cell dose, and immune rejection. Through in vitro and in vivo experiments, we also discovered that the CXCL13/CXCR5 pathway mediated the homing of DPCs and DSCs into hair follicle niches. This study underscores the potential of cell-based therapies for hair loss by targeted delivery of DPCs and DSCs to their respective niches and sheds light on the intriguing concept that isolated mesenchymal stem cells can home back to their original niche microenvironment.

## Graphical abstract

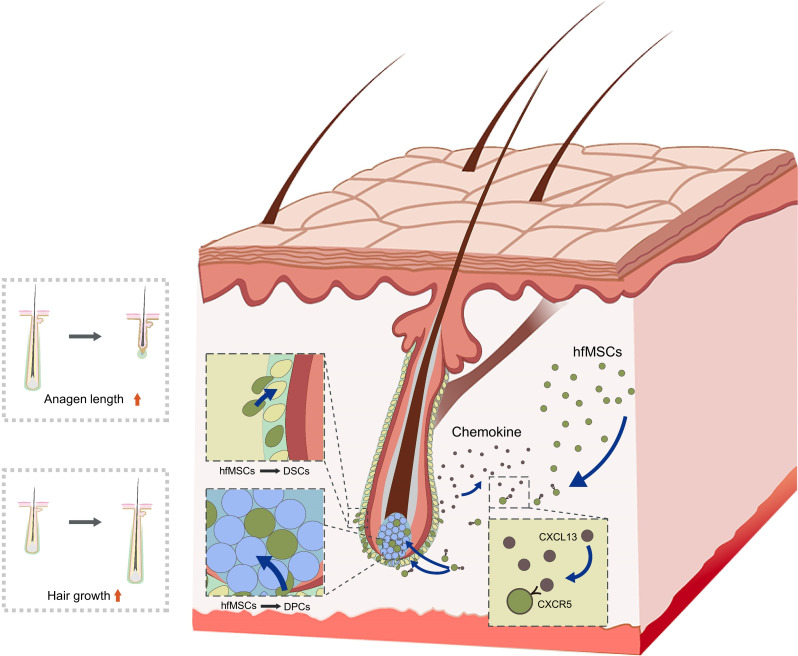

## Introduction

Hair loss, also known as alopecia or baldness, occurs when there are disruptions in the hair production cycle. It is characterized by shortened anagen phases and prolonged telogen phases of hair follicles (HFs), along with a gradual reduction in HF size (1–4). Although medication and hair transplantation are the mainstay treatments for hair loss (5–7), their effectiveness is limited. Medication works for certain patients only (8, 9) and may cause adverse effects, such as erectile dysfunction, skin rash, and itching (10–13). Hair transplantation, on the other hand, is hindered by the availability of donor hair grafts (14). Given these limitations, there is an urgent need to explore innovative therapeutic approaches for hair loss.

The dermal portion of an HF consists of 2 compartments: the dermal papilla (DP) and the dermal sheath (DS), both of which collectively house HF mesenchymal stem cells (hfMSCs). The progressive depletion or atrophy of dermal papilla cells (DPCs) is strongly associated with hair loss (15). Located at the base of the HF, the DP lies within a unique tissue surrounded by epithelial matrix cells. Studies have shown a positive correlation between the size of the DP and the dimensions of the follicle during the anagen phase (16). DPCs provide the instructive signals necessary for activating epithelial progenitors and orchestrating the fate of the HF, including the transition from anagen to telogen (17, 18). Consequently, DPCs are vital elements for regenerating and promoting hair growth. The DS, which encircles the outer layer of the HF, represents another dermal compartment. During the anagen phase, dermal sheath cells (DSCs) can be recruited to replenish DPCs and promote hair growth and cycling (19). Therefore, augmenting the numbers of DPCs and DSCs represents a promising therapeutic strategy for hair loss.

Stem cell therapy has emerged as a widely used approach for treating various diseases, and the therapeutic outcome depends on the efficient homing of transplanted cells that can self-renew and differentiate into niche cells essential for stem cell function (20–22). DPCs and DSCs are readily accessible and abundant sources of autologous cells containing numerous mesenchymal stem cells (MSCs) (23, 24), which suggests transplantation of these cells may be a promising therapeutic strategy for hair loss. However, the homing behavior of these cells has yet to be systematically investigated. The aims of this study were to investigate the homing phenomenon of exogenous DPCs and DSCs into HF niches and identify the pathway responsible for their migration. The results of this study will serve as a foundation for developing cell-based therapies for hair loss and reveal the remarkable capacity of MSCs to home back to their original niches.

## Results

### Exogenous hfMSCs migrate to HF niches and express niche cell markers.

**Figure 1:**
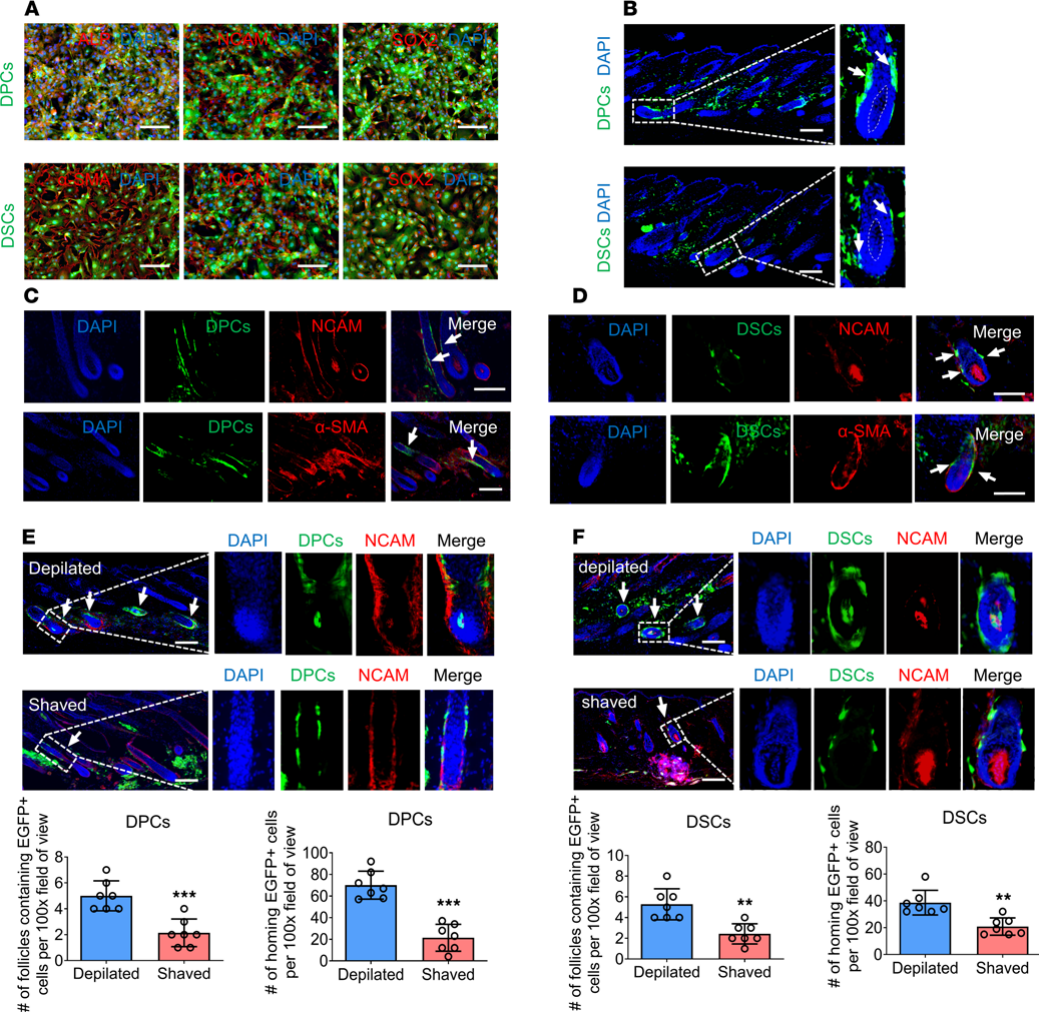
Exogenous hfMSCs home to HF niches, and depilation increases the homing rate. (**A**) Immunostaining for alkaline phosphatase, NCAM, and SOX2 in third-passage DPCs and for α-SMA, NCAM, and SOX2 in third-passage DSCs (representative of 3 experiments). (**B**) Representative HFs containing exogenous DPCs (arrow) or DSCs (arrow) 2 weeks after injection. Hatched lines denoted the DP (representative of 3 experiments). (**C** and **D**) Immunostaining for NCAM and α-SMA in HFs containing exogenous DPCs (arrow) or DSCs (arrow) (representative of 3 experiments). (**E** and **F**) HFs containing exogenous DPCs (arrow) or DSCs (arrow) were immunostained for NCAM 2 weeks after injection. The number of HFs containing EGFP^+^ cells and number of homing EGFP^+^ cells per ×100 original magnification field of view (*n* = 7 skin sections from 4 mice per group). Scale bars: 100 μm. Two-tailed Student’s *t* test. Data reported as mean ± SD. **P < 0.01, ***P < 0.001.

To investigate the ability of exogenous hfMSCs to home to their niches in the HF, we initially expanded DPCs and DSCs derived from vibrissa follicles of 5- to 6-week-old EGFP-transgenic mice up to passage 3. Immunostaining experiments demonstrated that DPCs expressed the DPC markers alkaline phosphatase, neural cell adhesion molecule (NCAM), lymphoid enhancer-binding factor 1, and leptin receptor, as well as the stem cell marker SRY-box transcription factor 2 (SOX2). Similarly, DSCs exhibited high expression of the DSC markers smooth muscle actin (α-SMA) and NCAM, along with SOX2 (Figure 1A and Supplemental Figure 2A; supplemental material available online with this article; https://doi.org/10.1172/jci.insight.173549DS1). Flow cytometry analysis showed that both DPCs and DSCs expressed the MSC-positive markers CD90, CD29, CD44, and stem cell antigen-1, and they weakly expressed the MSC-negative marker CD45 (Supplemental Figure 1B) (25). These findings suggested that DPCs and DSCs at passage 3 retained their identity and exhibited MSC properties.

Subsequently, we injected either DPCs or DSCs into the shaved dorsal skin of adult NOD/SCID mice and analyzed them 2 weeks later. Our results revealed that some of the exogenous DPCs and DSCs integrated into the DS follicle niche, which expressed the DSC markers NCAM and α-SMA. However, we did not observe any exogenous DPCs or DSCs within the DP of the shaved recipient area (Figure 1, B–D).

### Depilation enhances the migration of exogenous hfMSCs to HF niches.

**Figure 2:**
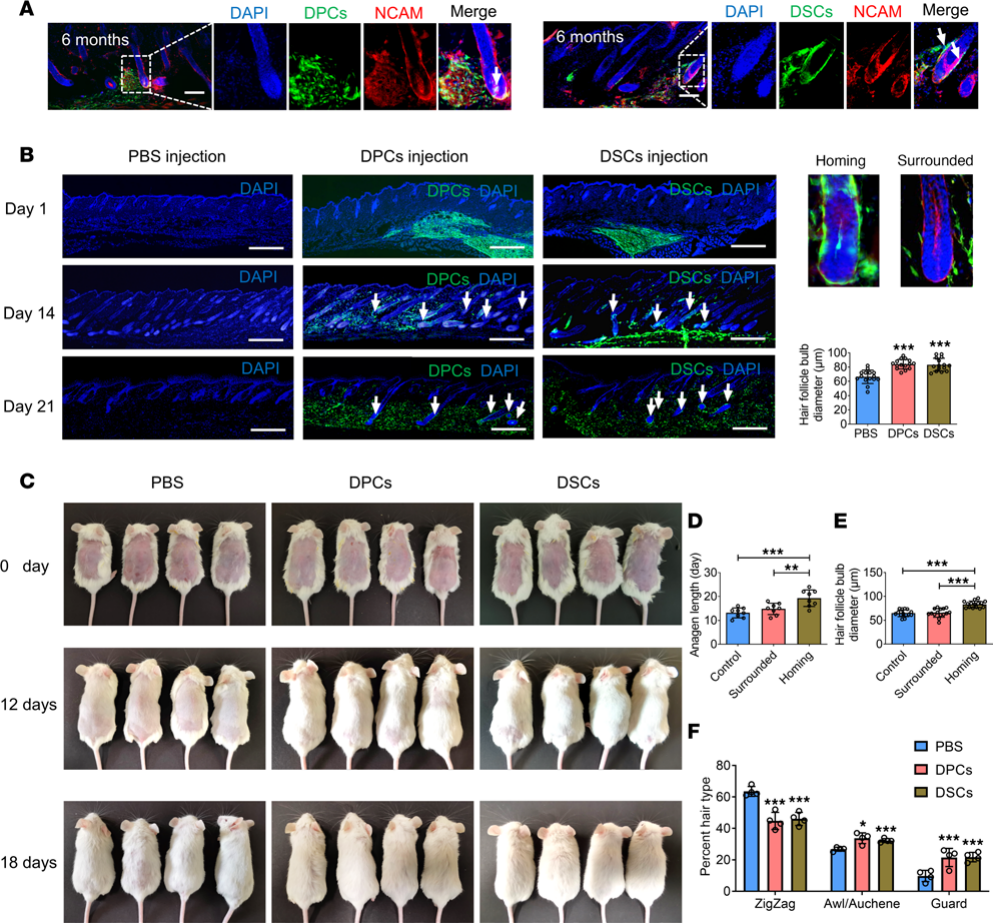
Exogenous hfMSCs promote HF growth via homing to HF niches. (**A**) HFs containing exogenous DPCs (arrow) or DSCs (arrow) were immunostained for NCAM at 6 months after injection (representative of 3 experiments). (**B**) Dorsal skin sections of mice injected with DPCs, DSCs, or PBS; arrows denoted the HFs containing exogenous DPCs or DSCs. Diameter of hair bulbs (*n* = 15 skin sections from 4 mice per group). (**C**) The hair growth of recipient mice. (**D** and **E**) Anagen length and hair bulb diameter of HFs containing exogenous DPCs/DSCs, HFs merely surrounded by exogenous DPCs/DSCs and HFs in PBS group (*n* = 8 skin sections from 4 mice per group in **D**; *n* = 15 skin sections from 4 mice per group in **E**). (**F**) Percentage of hairs of each type in mice of the PBS, DPC, and DSC groups 12 days after depilation (*n* = 4 mice per group). Scale bar: 100 μm in **A**; 400 μm in **B**. One-way ANOVA followed by Bonferroni’s post hoc test. Data reported as mean ± SD. *P < 0.05, **P < 0.01, ***P < 0.001.

A previous study has reported that depilation increases the recruitment of exogenous skin-derived precursors (SKPs) to HF niches (26). Inspired by this, we investigated if hfMSCs exhibit similar characteristics. To test this hypothesis, we depilated or shaved the dorsal skin of NOD/SCID mice before injecting DPCs and DSCs. Our results demonstrated that several DPCs and DSCs integrated into the DP and DS follicle niches in depilated mice, whereas neither DPCs nor DSCs integrated into the DP follicle niche in shaved mice. The depilated group not only had more follicles containing exogenous cells but also exhibited a higher number of homing cells compared with the shaved group (Figure 1, E and F). Further analysis revealed that exogenous DPCs and DSCs within the DP follicle niche expressed the DPC marker alkaline phosphatase (Supplemental Figure 2B). Last, we monitored the mice for up to 6 months and found that the homing DPCs and DSCs persisted within HF niches throughout the entire period (Figure 2A). Additionally, immunostaining revealed the presence of SOX2^+^EGFP^+^ cells within these HF niches (Supplemental Figure 2C). These findings suggest that homing DPCs and DSCs not only remain within the HF niche but also acquire specialized functions of niche cells.

To investigate whether depilation alters the homing behavior of exogenous DPCs and DSCs by affecting the HF cycle, we injected DPCs or DSCs into shaved NOD/SCID mouse dorsal skin at both the anagen and telogen stages without applying any extra mechanical depilation stimulation. Surprisingly, our results showed that DPCs and DSCs were only integrated into the DS follicle niche of the shaved mice in both groups, with no detectable integration into the DP. Furthermore, there was no significant difference in the number of follicles containing exogenous DPCs and/or DSCs (hereafter, DPCs/DSCs) or the number of homing DPCs/DSCs between both groups (Supplemental Figure 3). These findings suggest that anagen and telogen stages have limited impact on the migration behavior of DPCs and DSCs. Therefore, we speculate that depilation induces the homing of exogenous DPCs and DSCs through mechanical stimulation rather than by altering the HF cycle.

### Exogenous hfMSCs promote HF growth via homing to HF niches.

In consideration of the known correlation between DP size and follicle size (27), we investigated the potential of exogenous DPCs and DSCs to stimulate hair regeneration by homing to DP and DS niches. To address this, we administered injections of exogenous DPCs, exogenous DSCs, or PBS into adult NOD/SCID mice and analyzed the subsequent effects at 1, 14, and 21 days after injection. HFs in the DPC and DSC groups exhibited more robust growth than those in the PBS group (Figure 2, B and C). Additionally, HFs containing exogenous cells had the largest diameter and most prolonged anagen phase, followed by HFs encircled by exogenous cells (Figure 2, D and E). Notably, 12 days after depilation, mice in the DPC and DSC groups had a higher percentage of guard and awl or auchene hairs as well as a lower percentage of zigzag hairs, suggesting a more favorable hair type distribution compared with the PBS group (Figure 2F).

To eliminate potential confounding effects resulting from signaling molecules secreted by fibroblasts after injection, we incorporated an additional group of adult NOD/SCID mice that were injected with non-DPC, non-DSC dermal fibroblasts, in addition to those receiving exogenous DPCs and DSCs. The DPC and DSC groups exhibited enhanced hair growth compared with the fibroblast group. Specifically, at the 12-day time point after injection, mice in the DPC and DSC groups had a higher percentage of guard and awl or auchene hairs, coupled with a lower percentage of zigzag hairs, when compared with the fibroblast group (Supplemental Figure 4A). These results suggest that DPCs and DSCs elicit more potent stimulation of hair growth than do non-DPC, non-DSC dermal fibroblasts.

**Figure 3:**
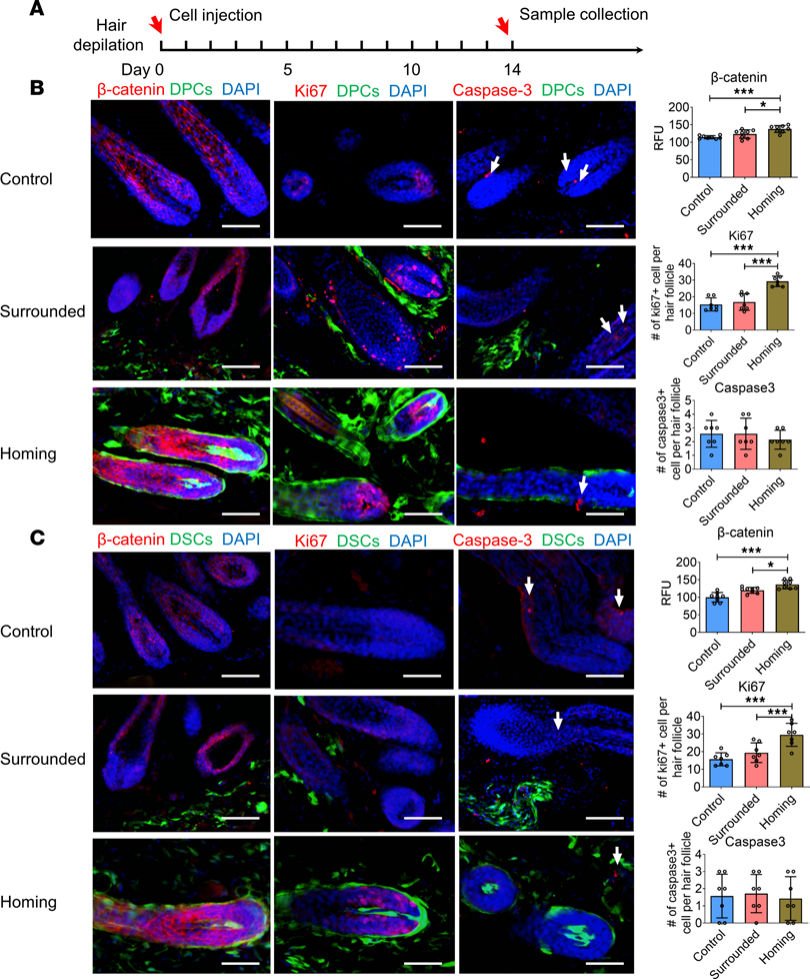
Exogenous hfMSCs promote the expression of β-catenin and Ki67 of the recipient HFs. (**A**) A schematic representing time points of sample collection. (**B** and **C**) Immunostaining for β-catenin, Ki67, and caspase-3 (arrow) in dorsal skin sections. These sections included HFs containing exogenous DPCs/DSCs, HFs merely surrounded by exogenous DPCs/DSCs and HFs in the PBS group. Analysis of β-catenin, Ki67, and caspase-3 expression in mouse skin sections (*n* = 7–8 skin sections from 5 mice per group). Scale bars: 50 μm. One-way ANOVA followed by Bonferroni’s post hoc test. Data reported as mean ± SD. *P < 0.05, ***P < 0.001. RFU, relative fluorescence units.

On day 14, we collected skin samples (Figure 3A) and conducted immunostaining for the hair growth marker β-catenin (28) and proliferation marker Ki67. These experiments revealed that HFs containing homing cells expressed the highest levels of β-catenin and Ki67, followed by HFs surrounded by exogenous DPCs/DSCs. In contrast, HFs in the PBS group had the least expression. Notably, there were no significant differences in the expression of the apoptosis marker caspase-3 among the 3 groups (Figure 3, B and C). These results indicate that exogenous DPCs and DSCs primarily promote hair growth by homing to HF niches, stimulating cell proliferation rather than reducing apoptosis.

### Cell passage, immunological response, and cell dose affect the homing behavior of exogenous hfMSCs.

**Figure 4:**
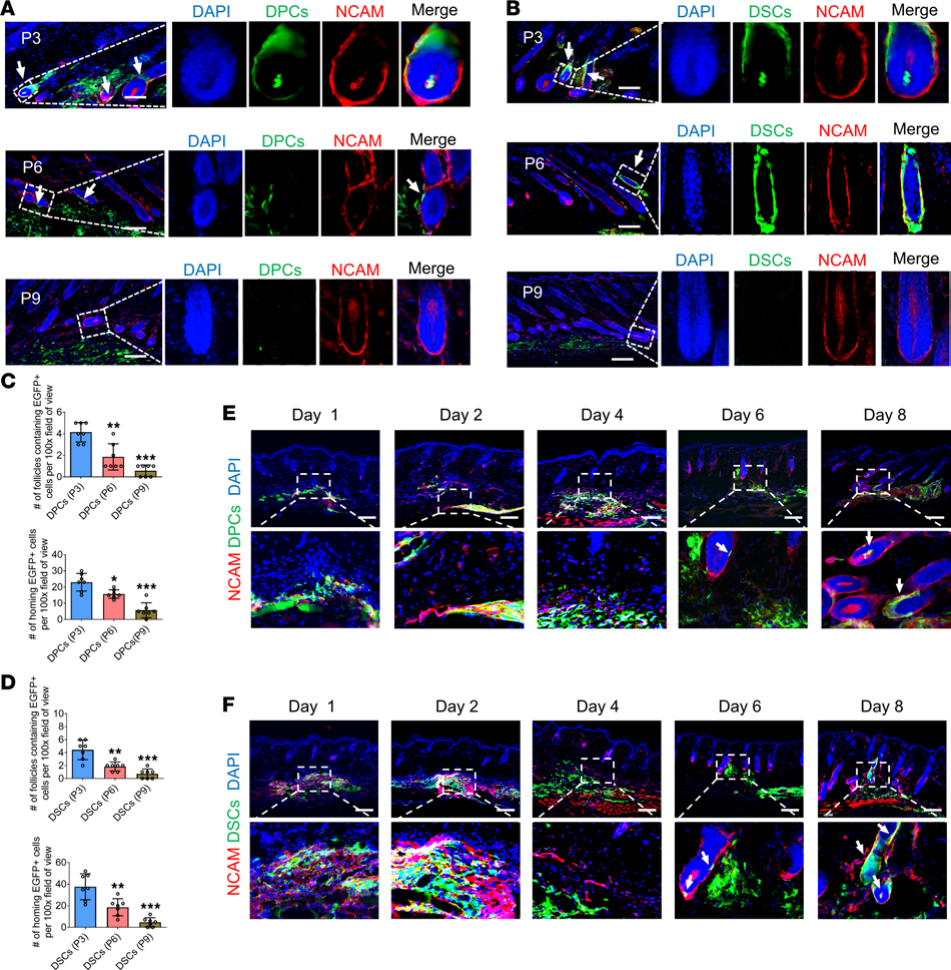
The migratory axis of the homing of exogenous hfMSCs is affected by cell passage. (**A** and **B**) Dorsal skin sections were harvested 2 weeks after intradermal injection of DPCs and DSCs at passages 3, 6, and 9 into mouse dorsal skin. Immunostaining showed the expression of NCAM (red) in HFs containing exogenous DPCs (green; arrow) and DSCs (green; arrow). (**C** and **D**) Number of HFs containing EGFP^+^ transplanted cells and number of homing EGFP^+^ cells per ×100 original magnification field of view as shown in **A** and **B** (*n* = 7 skin sections from 3 mice per group). One-way ANOVA followed by Bonferroni’s post hoc test. Data reported as mean ± SD. *P < 0.05, **P < 0.01, ***P < 0.001. (**E** and **F**) The migratory axis of DPCs (green) and DSCs (green). Arrows denote cells integrating into HF niches (representative of 3 experiments). Scale bars: 100 μm.

Serial passage is a common technique used to expand stem cells, but the migration ability and function of these cells may decline with increased passage (29). In this study, we evaluated the effects of serial passage on the homing behavior of DPCs and DSCs. We injected DPCs and DSCs at passages 3, 6, and 9 into the dorsal skin of adult NOD/SCID mice and analyzed the results after 2 weeks. Compared with cells at passage 3, DPCs and DSCs at passage 6 had fewer homing cells within HF niches, whereas those at passage 9 had the fewest homing cells (Figure 4, A–D).

**Figure 5:**
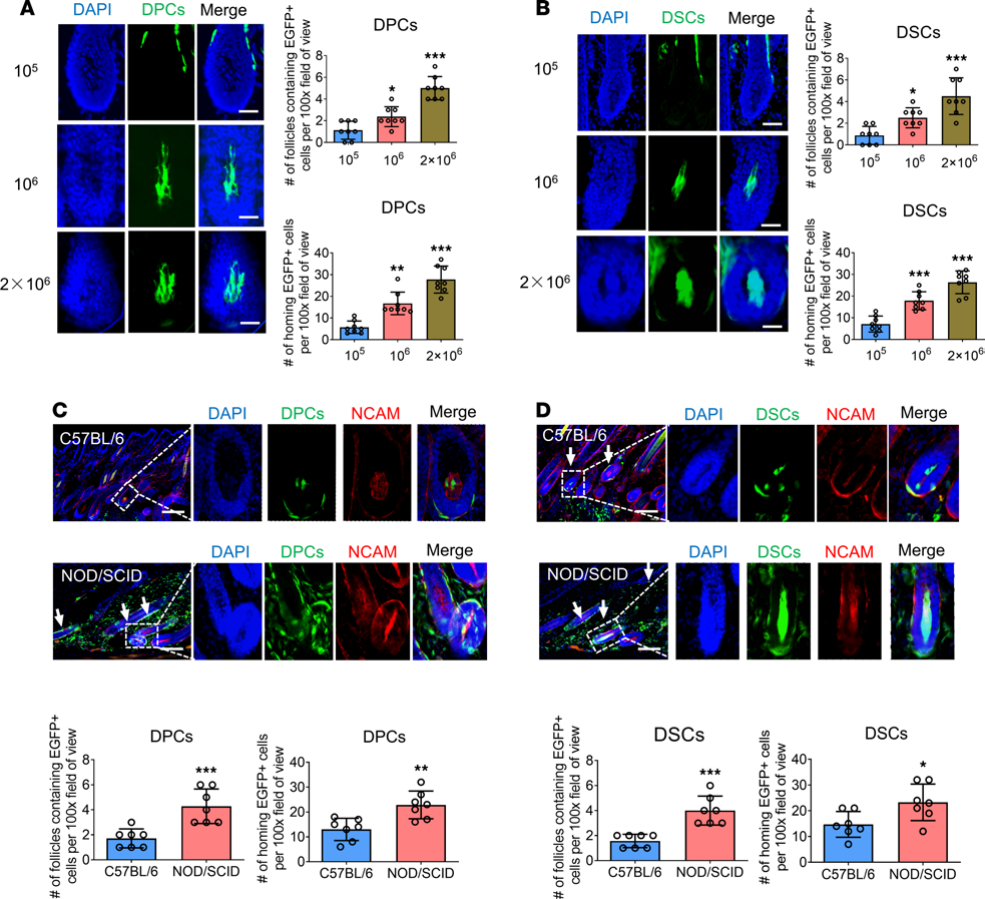
Cell dose and immunological rejection affect the homing rate of exogenous hfMSCs. (**A** and **B**) Longitudinal sections of HF were harvested 2 weeks after intradermal injection of DPCs and DSCs at different doses into NOD/SCID mouse skin. Number of HFs containing EGFP^+^ transplanted cells and number of homing EGFP^+^ cells per ×100 original magnification field of view (*n* = 8 skin sections from 4 mice per group). (**C** and **D**) Dorsal skin sections were harvested 2 weeks after intradermal injection of DPCs and DSCs into C57BL/6 and NOD/SCID mouse skin. HFs containing exogenous DPCs (green; arrow) or DSCs (green; arrow) were immunostained for NCAM (red). Number of HFs containing EGFP^+^ transplanted cells and number of homing EGFP^+^ cells per ×100 original magnification field of view (*n* = 7 skin sections from 4 mice per group). Scale bars: 25 μm in **A** and **B**; 100 μm in **C** and **D**. Statistical comparisons were performed using 1-way ANOVA followed by Bonferroni’s post hoc test (**A** and **B**) and 2-tailed Student’s *t* test (**C** and **D**). Data reported as mean ± SD. *P < 0.05, **P < 0.01, ***P < 0.001.

To further investigate whether the dose of transplanted cells affected their homing behavior, we injected DPCs/DSCs into depilated NOD/SCID mouse dorsal skin at doses of 1 × 10^5^, 1 × 10^6^, and 2 × 10^6^ cells and analyzed the results after 2 weeks. Remarkably, the number of follicles containing exogenous DPCs/DSCs and the integration of exogenous DPCs/DSCs into the HF niches were positively correlated with the transplanted cell doses (Figure 5, A and B).

To examine the potential influence of immunological rejection on the homing of exogenous DPCs/DSCs into HF niches, we injected these cells into depilated C57BL/6J mice and NOD/SCID mice dorsal skin and compared the results. Our analysis revealed a significant reduction in the number of follicles containing exogenous DPCs/DSCs and the number of exogenous DPCs/DSCs that integrated into the HF niches within the C57BL/6J mouse group when compared with the NOD/SCID mice (Figure 5, C and D).

To gain further insights, we performed immunofluorescence staining using specific markers for macrophages (F4/80), T cells (CD4, CD8), and inflammatory factors (IL-6, TNF-α). There was a remarkable increase in the abundance of these immune cell populations and inflammatory factors surrounding the injected cells in the C57BL/6J mouse model, which starkly contrasted with the observations made in the NOD/SCID mouse model (Supplemental Figure 2E). Additionally, our analysis detected a noteworthy elevation in the number of apoptotic cells (caspase-3^+^) within the injected cell population in the C57BL/6J mice, relative to that observed in NOD/SCID mice (Supplemental Figure 2D). These findings suggest that immunological rejection compromises the migratory capabilities of DPCs and DSCs.

**Figure 6:**
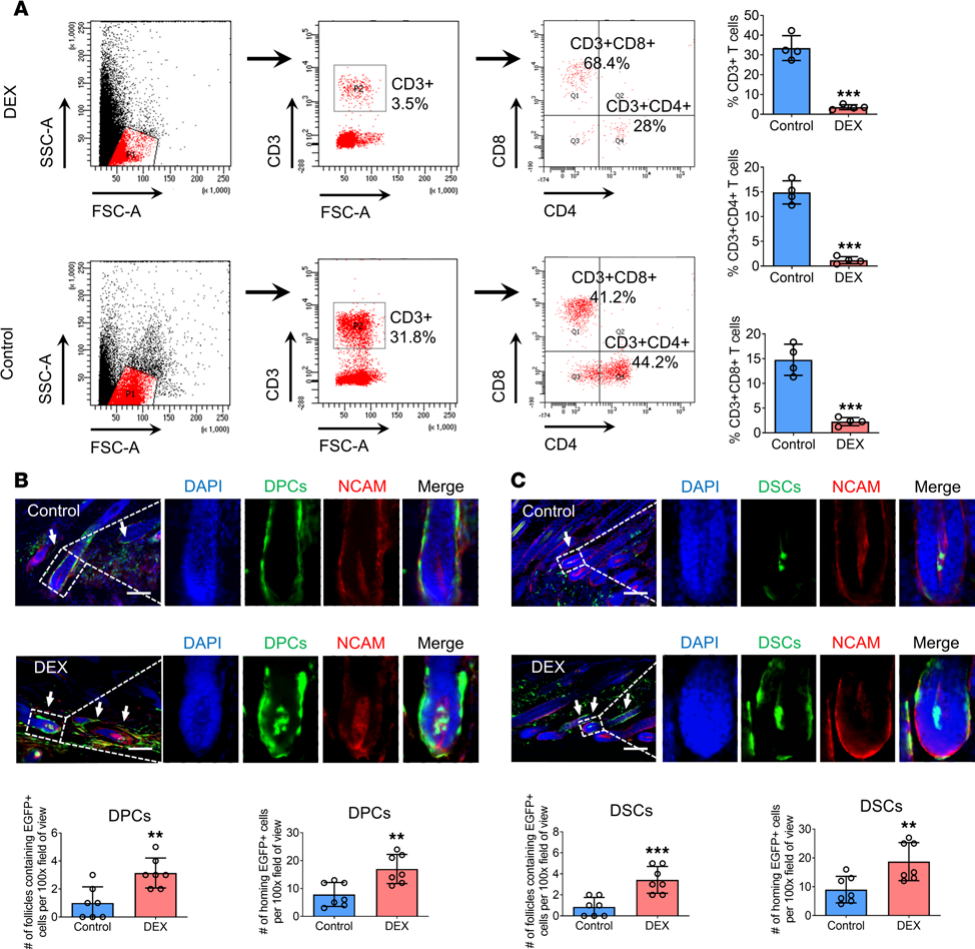
Dexamethasone restores the homing rate of exogenous hfMSCs impaired by immunological rejection. (**A**) Flow cytometry detected the percentages of CD3^+^, CD3^+^CD4^+^, and CD3^+^CD8^+^ T cells in the blood of C57BL/6J mice after treatment with dexamethasone (*n* = 4 mice per group). (**B** and **C**) Longitudinal sections of HF were harvested 2 weeks after intradermal injection of DPCs and DSCs into dexamethasone-treated and vehicle-treated C57BL/6J mice. Immunostaining showed the expression of NCAM (red) in HFs containing exogenous DPCs (green; arrow) and DSCs (green; arrow). Number of HFs containing EGFP^+^ transplanted cells and number of homing EGFP^+^ cells per ×100 original magnification field of view (*n* = 7 skin sections from 4 mice per group). Scale bars: 100 μm. Two-tailed Student’s *t* test. Data reported as mean ± SD. **P < 0.01, ***P < 0.001.

To further validate the effect of immunological rejection on the homing of DPCs/DSCs, we established an immunosuppressive model in C57BL/6J mice via i.p. injection of dexamethasone (30). Flow cytometry analysis revealed that dexamethasone treatment resulted in a significant decrease in the numbers of CD3^+^ T cells, CD3^+^CD4^+^ T cells, and CD3^+^CD8^+^ T cells in the blood of C57BL/6J mice (Figure 6A). Subsequently, we transplanted DPCs/DSCs into the depilated dorsal skin of both dexamethasone-treated and vehicle-treated C57BL/6J mice. We observed a marked increase in the number of follicles containing exogenous DPCs/DSCs as well as in the number of exogenous DPCs/DSCs integrated into the HF niches in dexamethasone-treated mice compared with those in vehicle-treated mice (Figure 6, B and C). These findings support the notion that immunological rejection impairs the homing ability of exogenous DPCs/DSCs into HF niches.

### The molecular mechanisms underlying the effects of cell passage on homing rates.

**Figure 7:**
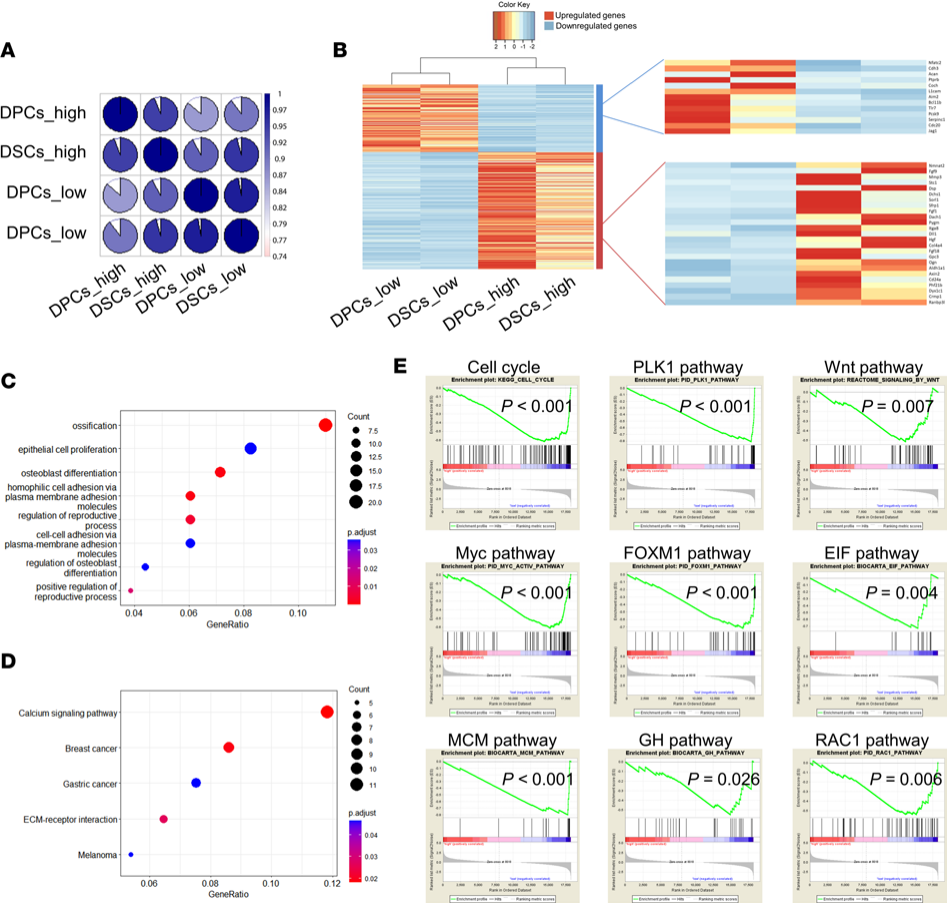
The molecular mechanism underlying phenotypic differences between low-passage and high-passage DPCs/DSCs. (**A**) Spearman’s rank correlations were computed for every pair of microarray experiments. The resulting color-coded correlation matrix revealed the transcriptomal similarities among low-passage and high-passage DPCs/DSCs (the similarities are demonstrated by pie chart proportion). (**B**) The heatmap shows the relative expression levels of 363 differential genes between low-passage and high-passage DPCs/DSCs. Relative expression levels are color-coded (see the color key). The cluster analysis above the heatmap shows that low-passage DPCs/DSCs were more related to each other than they were to high-passage DPCs/DSCs. Differential genes associated with cell proliferation and stemness. (**C**) Gene ontology and (**D**) Kyoto Encyclopedia of Genes and Genomes pathway enrichment analysis of the differential genes between low-passage and high-passage DPCs/DSCs. (**E**) Gene set enrichment analysis demonstrated the pathways inhibited in high-passage DPCs/DSCs.

To uncover the molecular mechanisms underlying the difference in homing rate between low-passage (passage 3) and high-passage (passage 9) DPCs/DSCs, we conducted a global gene expression analysis, comparing gene expression between these cell types (Gene Expression Omnibus [GEO] GSE236091). Our Spearman’s rank correlation analysis showed that gene expression profiles between low-passage DPCs and low-passage DSCs were very similar, with intersample correlations of 0.97, whereas the sample correlations between low-passage and high-passage DPCs/DSCs were relatively different, ranging from 0.86 to 0.95 (Figure 7A).

Our analysis of differential gene expression revealed that the expression of 363 genes was upregulated (230 genes; Supplemental Table 4) or downregulated (133 genes; Supplemental Table 5) at least 4-fold in high-passage DPCs/DSCs. Cluster analysis showed that low-passage DPCs/DSCs were more related to each other than to high-passage DPCs/DSCs. The upregulated genes included inhibitors of the Wnt (*Sfrp1*, *Axin2*) and BMP pathways (*Ranbp3l*), as well as inhibitors of cell proliferation and stem cell differentiation (*Mmp3*, *Crmp1*, *Dach1*). The downregulated genes included several genes that regulate cell stemness (*Nfatc2*, *Cdh3*, *L1cam*, *Jag1*; Figure 7B). Therefore, the reduction of homing rates in high-passage DPCs/DSCs may be associated with the inhibition of the Wnt and BMP pathways and inactivation of genes that regulate cell stemness and proliferation.

Subsequently, we performed gene ontology and Kyoto Encyclopedia of Genes and Genomes pathway enrichment analysis of the differentially expressed genes between low-passage and high-passage DPCs/DSCs. The gene ontology enrichment analysis predominantly categorized differentially expressed genes as being involved in ossification, osteoblast differentiation, homophilic cell adhesion, and regulation of the reproductive process (Figure 7C). The Kyoto Encyclopedia of Genes and Genomes pathway enrichment primarily identified the calcium signaling, breast cancer, and extracellular matrix–receptor interaction pathways (Figure 7D).

Furthermore, we applied gene set enrichment analysis to determine the biological pathways that contributed to the phenotypic differences between low-passage and high-passage DPCs/DSCs. Remarkably, the results demonstrated enrichment of genes related to the cell cycle and pathways relevant to stem cell phenotypes, including the PLK1, Wnt, Myc, FOXM1, EIF, MCM, GH, and RAC1 pathways (Figure 7E). Consequently, on the basis of these findings, we propose that the inhibition of PLK1, Wnt, Myc, FOXM1, EIF, MCM, GH, and RAC1 pathways may account for the reduced homing rate observed in high-passage DPCs/DSCs.

### The CXCL13/CXCR5 axis regulates the depilation-induced homing of exogenous hfMSCs into follicle niches.

To uncover the mechanism underlying depilation-induced homing of exogenous DPCs and DSCs, we conducted a comprehensive analysis of their migration and integration into follicle niches after injection into depilated NOD/SCID mouse dorsal skin. Our findings demonstrate that exogenous DPCs and DSCs gradually migrated toward HF niches and first integrated into the DS on day 6 (Figure 4, E and F), coinciding with obvious morphological changes in the HFs (Supplemental Figure 4B). These findings suggest that follicles provide instructive signals that trigger the migration of DPC and DSC prior to day 6.

**Figure 8:**
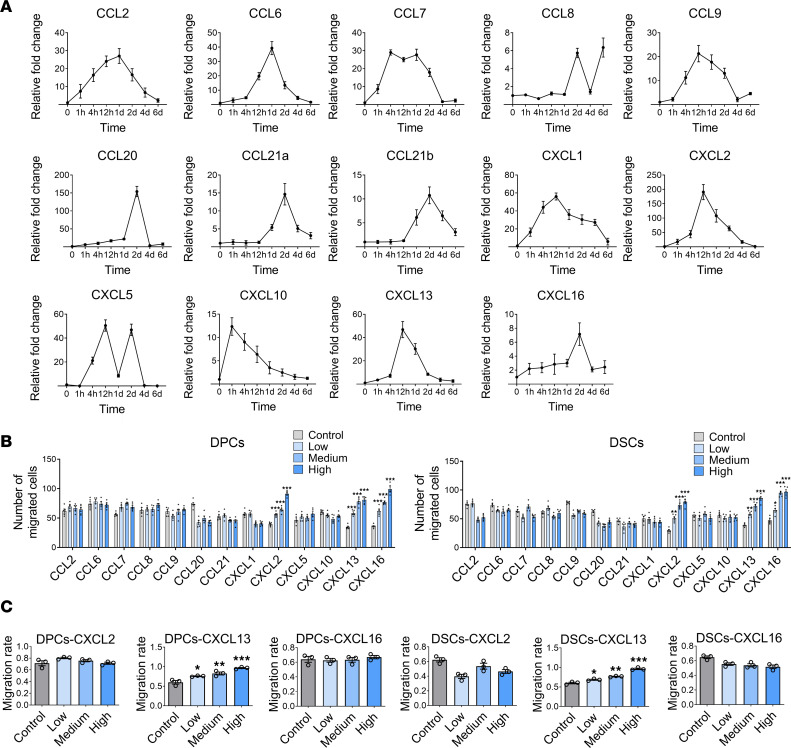
Chemokine mediates the homing of exogenous hfMSCs into follicle niches. (**A**) RT-qPCR experiments showed the upregulated chemokines after depilation of NOD/SCID dorsal skin (*n* = 3). (**B**) Transwell chemotaxis assays were performed to detect the migratory response of DPCs and DSCs to chemokines at different concentrations (namely, control: 0 ng/mL; low: 5 ng/mL; medium: 50 ng/mL; and high: 500 ng/mL) (*n* = 5). (**C**) Wound healing assays were performed to detect the chemotaxis effects of CXCL2, CXCL13, and CXCL16 on DPCs and DSCs (concentrations are the same as in **B**) (*n* = 3). One-way ANOVA followed by Bonferroni’s post hoc test. Data reported as mean ± SD. *P < 0.05, **P < 0.01, ***P < 0.001.

To further elucidate the involved signaling mechanisms, we performed a global gene expression analysis of depilated NOD/SCID skin samples at different times after depilation (GEO GSE208551). The results showed time-dependent upregulation of several chemokines, including CCL2, CCL6, CCL8, CCL9, CCL20, CCL21a, CCL21b, CXCL1, CXCL2, CXCL5, CXCL10, CXCL13, and CXCL16. Reverse transcription quantitative PCR (RT-qPCR) experiments verified the increased mRNA expression of these chemokines (Figure 8A), implying their critical role in regulating the homing of DPCs and DSCs to follicle niches.

**Figure 9:**
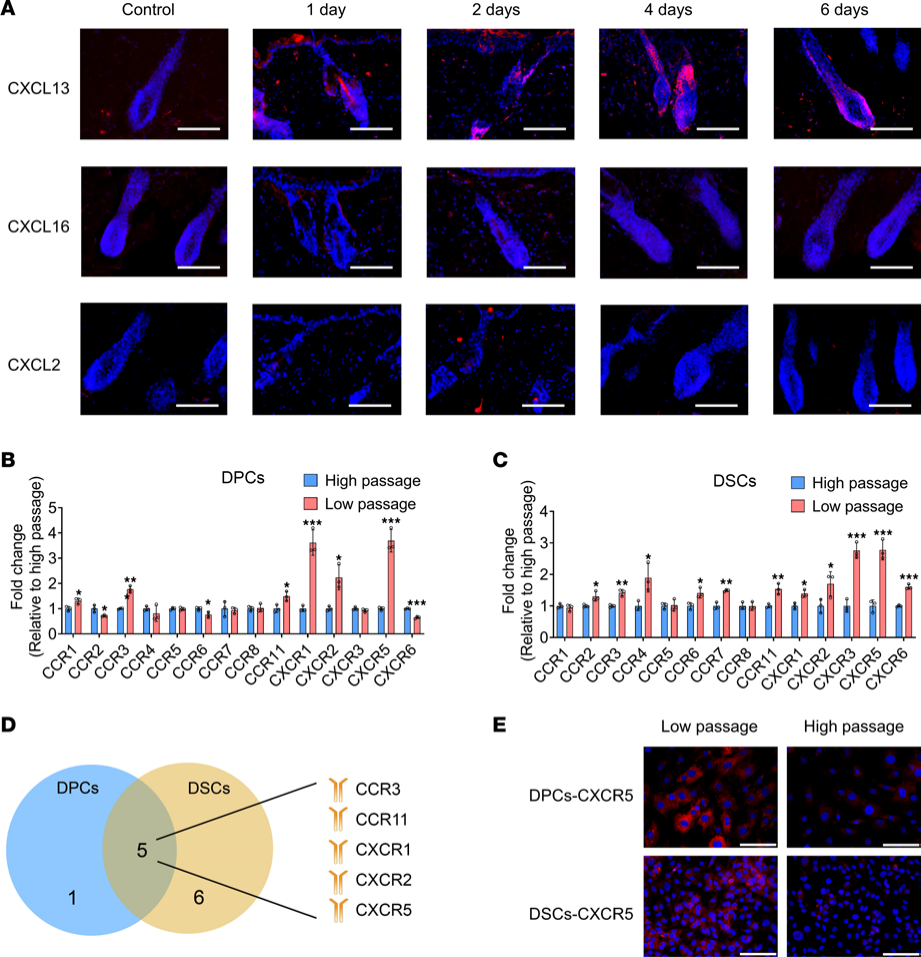
Chemokine receptor mediates the homing of exogenous hfMSCs into follicle niches. (**A**) Dorsal skin sections were harvested 1, 2, 4, and 6 days after depilation. (The control group comprised a section of shaved dorsal skin.) Immunostaining showed the expression of CXCL13, CXCL16, and CXCL2 (representative of 3 experiments). (**B** and **C**) RT-qPCR experiments showed the expression of receptors related to upregulated chemokines in low-passage and high-passage DPCs/DSCs (*n* = 3). Two-tailed Student’s *t* test. Data reported as mean ± SD. *P < 0.05, **P < 0.01, ***P < 0.001. (**D**) Venn diagram shows the intersection of the downregulated chemokine receptors in high-passage DPCs/DSCs. (**E**) Immunostaining shows the expression of CXCR5 in low-passage and high-passage DPCs/DSCs (representative of 3 experiments). Scale bars: 100 μm in **A** and **E**.

Transwell chemotaxis assays were performed to test the migratory response of DPCs and DSCs to the upregulated chemokines. Chemokines were added to the lower part of the Transwell chamber at different concentrations (control, 0 ng/mL; low, 5 ng/mL; medium, 50 ng/mL; high, 500 ng/mL). Among the chemokines upregulated after skin depilation, only CXCL2, CXCL13, and CXCL16 stimulated DPCs and DSCs to penetrate the Transwell basement membrane. Moreover, the chemotactic effect was concentration dependent (Figure 8B and Supplemental Figures 5 and 6). Wound healing assays further verified that only CXCL13 significantly enhanced the migration of DPCs and DSCs in a concentration-dependent manner (Figure 8C and Supplemental Figures 7 and 8). Immunostaining revealed the overexpression of CXCL13 in HFs after depilation (Figure 9A), suggesting its critical role in regulating the homing of DPCs and DSCs into HF niches.

We then investigated the key chemokine receptors involved in this process. We hypothesized that the expression level of the key chemokine receptor would be downregulated in high-passage DPCs and DSCs, as the homing rate decreased with increasing passage. Our results showed substantial downregulation of CCR3, CCR11, CXCR1, CXCR2, and CXCR5 levels in high-passage DPCs and DSCs (Figure 9, B–D, and Supplemental Table 1). Interestingly, CXCR5 is the receptor of CXCL13, suggesting the crucial role of the CXCL13/CXCR5 axis in the homing of DPCs/DSCs into HF niches. Immunostaining analysis validated the decreased expression of CXCR5 in high-passage DPCs and DSCs (Figure 9E).

To investigate the interaction between CXCL13 and CXCR5, we conducted molecular docking analysis using protein structures obtained from the UniProt database. Pymol 2.3.0 and Hdock software were used for docking CXCR5 and CXCL13. Docking results showed a high binding affinity of –308.57 kcal/mol between CXCL13 and CXCR5, with hydrogen bonds formed by interacting residues. Key residues such as VAL-154, TYR-150, and ARG-162 in CXCR5 formed hydrogen bonds with TYR-27, LEU-23, and ILE-22 in CXCL13, respectively, with bond distances of 3.3 Å, 3.2 Å, and 2.9 Å, respectively (Supplemental Figure 9). These residues are likely to be important in the biological activity of the 2 proteins.

**Figure 10:**
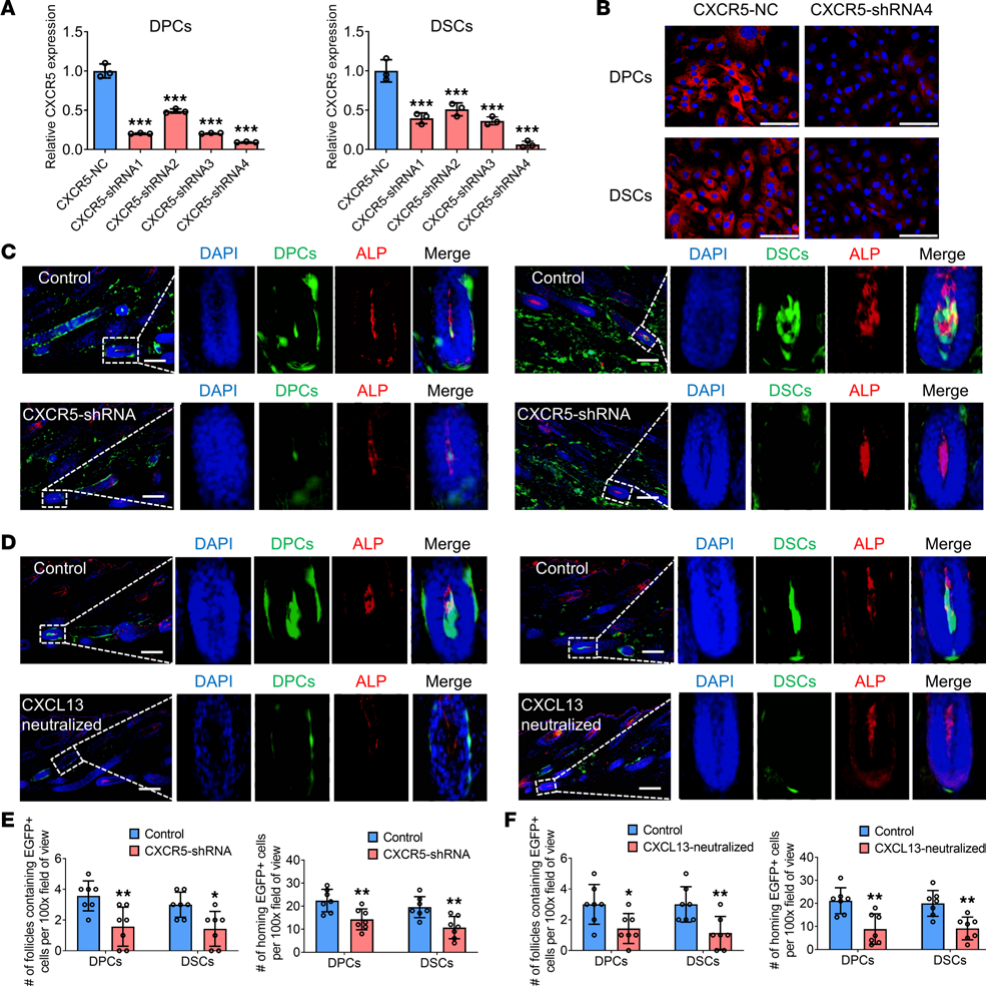
The CXCL13/CXCR5 axis mediates the homing of exogenous hfMSCs into follicle niches. (**A**) RT-qPCR experiments show the mRNA expression of CXCR5 in DPCs/DSCs after transfection with lentiviral vector expressing CXCR5 shRNA (*n* = 3). (**B**) Immunostaining shows the protein expression of CXCR5 in DPCs/DSCs after transfection with lentiviral vector expressing CXCR5 shRNA4 (representative of 3 experiments). (**C**) DPCs/DSCs transfected with lentiviral vector expressing CXCR5-shRNA4 were injected into the dorsal skin of depilated NOD/SCID mice. (**D**) DPCs/DSCs mixed with CXCL13 neutralizing Ab were injected into the dorsal skin of depilated NOD/SCID mice. HFs containing exogenous DPCs (green; arrow) or DSCs (green; arrow) were immunostained for alkaline phosphatase (ALP) (red) (**C** and **D**). (**E** and **F**) Number of HFs containing EGFP^+^ transplanted cells and number of homing EGFP^+^ cells per ×100 original magnification field of view, as shown in **C** and **D** (*n* = 7 skin sections from 4 mice per group). Statistical comparisons were performed using 1-way ANOVA followed by Bonferroni’s post hoc test (**A**) and 2-tailed Student’s *t* test (**E** and **F**). Scale bars: 100 μm. Data are reported as mean ± SD. *P < 0.05, **P < 0.01, ***P < 0.001.

To verify the role of CXCL13/CXCR5 axis in the homing of DPCs and DSCs, we investigated the effects of CXCR5 knockdown or CXCL13 neutralization in vivo. We used 4 CXCR5 shRNA plasmids to knock down CXCR5 expression in DPCs and DSCs. RT-qPCR analysis demonstrated that the highest knockdown efficiency was with CXCR5-shRNA4 (Figure 10A). Immunostaining verified substantial downregulation of CXCR5 expression in DPCs and DSCs after transfection with CXCR5 shRNA4 (Figure 10B). Furthermore, intradermal injection of the transfected DPCs/DSCs into depilated NOD/SCID mice resulted in a significant decrease in the number of follicles containing exogenous DPCs/DSCs and their integration into HF niches (Figure 10, C and E). Additionally, intradermal injection of DPCs/DSCs mixed with CXCL13 neutralizing Ab into depilated NOD/SCID mice led to a substantial reduction in the number of follicles containing exogenous DPCs/DSCs and their integration into HF niches, compared with the control group (Figure 10, D and F). Taken together, these findings strongly support the critical role of the CXCL13/CXCR5 axis in regulating the homing process of DPCs/DSCs into HF niches.

## Discussion

The results of our study yield several key inferences. First, we have demonstrated that exogenous DPCs and DSCs can return to HF niches, express niche markers, and promote hair growth by increasing hair diameter, lengthening the anagen phase, and producing longer hair shafts. Second, depilation is a stimulant for the homing of DPCs and DSCs into follicle niches, a process regulated by the CXCL13/CXCR5 axis. Third, we observed that immunological rejection markedly influences the homing behavior of DPCs and DSCs, and the transplanted cells home to HF niches in a dose‑dependent manner. Last, we identified significant differences in homing rates between low-passage and high-passage DPCs and DSCs, which potentially can be attributed to the loss of cell stemness and downregulated expression of chemokine receptors in high-passage cultures.

In this study, we traced the migratory axis of transplanted DPCs/DSCs. Our findings indicate that these cells progressively migrated toward the resident HFs, integrating into them by day 6 after transplantation (Figure 4, E and F). Notably, the integrated HFs appeared to be fully developed, suggesting that DPCs/DSCs promote hair growth through integration with resident HFs rather than recruiting skin cells and initiating de novo HF generation. The exact mechanisms underlying the observed effects of DPCs and DSCs on hair growth remain unclear, requiring further investigations to elucidate their detailed action mechanisms for the development of more effective hair loss treatments.

Androgenetic alopecia (AGA) is a chronic and progressive hair loss disorder that affects up to 50% of men and women. The pathogenesis of AGA involves the interaction between androgens and androgen receptors (ARs) in the DPCs of balding scalp areas. The overexpression of ARs in these HFs results in increased sensitivity to androgens, leading to hair miniaturization and eventual loss (31, 32). We speculate that transplantation of DPCs and DSCs from nonbald areas with low AR expression could be a potential treatment for AGA. These cells may home to the affected HFs and reduce their androgen sensitivity. Overall, our findings provide compelling evidence supporting the feasibility of using DPCs and DSCs from nonbalding regions as a potential treatment for AGA. Clinical trials are warranted to verify the efficacy of this approach.

Cell therapy has been proposed as a promising approach for the treatment of various diseases. The efficiency of cell therapy depends on the properties of the donor cells, including their passage number. Previous studies have indicated that low-passage cells possess superior therapeutic potential compared with high-passage cells, because of their enhanced migration and homing abilities (33, 34). Consistent with these findings, our study demonstrates that low-passage cells have a significantly higher homing rate than do high-passage cells (Figure 4, A–D), and this could be attributed to the downregulation of chemokine receptors in high-passage cells (Figure 9, B and C). Our findings offer valuable insights into the mechanisms underlying the effects of cell passage on the therapeutic potential of DPCs and DSCs, suggesting that targeting chemokine receptors can improve their therapeutic potential.

DP and DS follicle niches are crucial for maintaining or inducing the hfMSCs phenotype. Research has demonstrated that the introduction of exogenous SKPs to these niches can lead to the expression of DPC and DSC markers, whereas SKPs integrated into the interfollicular dermis exhibit dermal fibroblast markers instead of SKP markers (26). This underscores the critical importance of the HF niches in determining both cell phenotype and fate. Notably, our study yielded similar results, with exogenous DPCs and DSCs homing to DP and DS niches that expressed DPC and DSC markers, whereas those remaining in the interfollicular dermis gradually lost their markers over time (Figure 1, C and D, and Supplemental Figure 2B). These findings provide compelling evidence of the crucial role played by HF niches in determining cell phenotype and fate.

In our study, we observed that follicles containing exogenous DPCs and DSCs were larger than those without these cells. Although an increased number of DPCs and DSCs may contribute to this finding, it is worth noting that the DPCs and DSCs used in our experiment were isolated from vibrissae rather than dorsal skin HFs. Vibrissae follicles possess unique characteristics, including a larger size and faster growth rate (35). These distinctive attributes suggest that vibrissae DPCs and DSCs may exhibit enhanced proliferative capacity and superior hair-inductive abilities when compared with cells from other sources. This difference in origin could potentially affect the observed size variation of the integrated HF. To further investigate this, studies could be conducted to compare the size of HFs after transplanting vibrissae DPCs/DSCs and dorsal DPCs/DSCs.

Wax-based depilation could provide an additional mechanical stimulus and lead to the destruction of a significant portion of the HF stem cell compartment, followed by rapid regeneration from the secondary hair germ (36). In a previous study, researchers demonstrated that hair plucking induces apoptosis in HF cells and stimulates the expression of CCL2 (37). Furthermore, various regions within the HF exhibit the expression of chemokines, including CCL2, CCL20, and CCL8, in response to mechanical stress (38). In our study, we observed that depilation induces the expression of CXCL13, which mediates the homing of injected DPCs and DSCs. Therefore, we speculate that depilation induces apoptosis in HF cells of NOD/SCID mice, resulting in the upregulation of CXCL13 expression and the recruitment of the injected DPCs and DSCs.

In this study, we observed impaired homing in immunocompetent C57BL/B6 mice. Several factors may contribute to this impairment. First, the immunogenicity of EGFP should be considered a potential factor. EGFP, being a foreign protein, can elicit immune responses in the host organism. Previous studies have documented concerns about the immunogenicity associated with GFP expression in certain mouse strains, including C57BL/6J (39, 40). These immune responses could potentially lead to inflammation or recruitment of immune cells at the site of GFP-expressing cells, consequently affecting their homing ability. Second, when injecting cells derived from the EGFP^+^ C57BL/6J mouse strain into immunocompetent C57BL/6J mice, it is possible that minor genetic differences or specific antigens within supposedly genetically identical C57BL/6J mouse strains may exist (41). Moreover, during in vitro culture, cells can undergo changes in antigenicity, due to the expression of new cell surface markers or secretion of proteins (42). These newly expressed molecules may be recognized as foreign antigens by the host immune system, triggering an immune rejection response upon injection into another individual.

Overall, our study highlights the potential of DPC/DSC–based cell therapy as a promising avenue for hair regeneration and provides insights into the molecular mechanisms underlying this process. We believe that more research in this area has the potential to lead to transformative breakthroughs in the field of regenerative medicine, with far-reaching implications for a range of diseases and conditions beyond hair loss.

## Methods

Additional information can be found in Supplemental Methods.

### Cell isolation and culture.

DPCs and DSCs were isolated from 5- to 6-week-old EGFP-transgenic C57BL/6J mouse (GemPharmatech) vibrissal follicles. The isolation process of DPCs and DSCs was as described previously, with minor modifications (43, 44). Briefly, vibrissal follicles were isolated from the cheeks of the mice and incubated in DMEM (Gibco). A combination of physical dissection and enzymatic digestion was used to isolate DPCs and DSCs. Initially, the hair bulb region and the DS portion (including the DS, hair shaft, and outer root sheath) of the HFs were separated using a physical dissection technique (Supplemental Figure 1A). To isolate the DPCs, hair bulbs were cut with scissors and incubated in 0.2% (wt/vol) collagenase I (Sigma) at 37°C for 2 hours. After centrifugation at 70*g* for 5 minutes at 20°C, the supernatant was discarded, and the cell pellet was resuspended in DMEM. DP spheres were isolated directly by careful pipetting using an inverted phase-contrast microscope (Supplemental Figure 1A), transferred to cell culture dishes, and cultured in DMEM supplemented with 10% (vol/vol) FBS (Gibco) at 37°C and 5% CO_2_. Explants were cultured for 7 days, and when the outgrowth became subconfluent, cells were harvested with 0.25% (wt/vol) trypsin-EDTA and subcultured at a ratio of 1:3.

To isolate the DSCs, the DS portion (including the DS, hair shaft, and outer root sheath) was treated with 0.1% (wt/vol) dispase II (Sigma) at 37°C for 30 minutes, and then the DS was separated from the HF shaft with forceps under a stereoscope (Supplemental Figure 1A). The isolated DS was incubated with 0.2% (wt/vol) collagenase I (Sigma) at 37°C for 2 hours. The suspension then was filtered with 70 μm strainers and centrifuged at 200*g* for 5 minutes at 20°C. After the supernatant was gently discarded, the cells were resuspended with appropriate medium volume of DMEM supplemented with 10% (vol/vol) FBS and were cultured at 37°C and 5% CO_2_.

### In vivo experiment.

For transplants, 10^5^ to 2 × 10^6^ passaged, dissociated murine DPCs and DSCs were intradermally injected into the skin of adult NOD/SCID mice (GemPharmatech) or C57BL/6J mice (GemPharmatech) immediately after depilation or shaving. Specifically, the injection was targeted at the dermis layer located above the panniculus carnosus muscle layer. For cell injection, the DPCs/DSCs were intradermally injected at 9 sites evenly distributed on the dorsal skin of the mice (an injection site pattern of 3 rows and 3 columns) (Figure 2C and Supplemental Figure 4A). Each site on the dorsal skin was intradermally injected with 2 × 10^6^ cells at a volume of 200 μL. For the neutralizing CXCL13 study, 50 μg/mL CXCL13 neutralizing Ab (R&D Systems, Bio-Techne; catalog AF470) was mixed with 2 × 10^6^ DPCs/DSCs, and the mixture was intradermally injected into depilated back skin of adult NOD/SCID mice. For dexamethasone treatment, 5 mg/kg/d dexamethasone (Sigma) was administered by i.p. injection for 2 weeks before cell injection. All animals were purchased from the Experimental Animal Centre at Southern Medical University (Guangzhou, China). All animal experiments were carried out with the approval of the Southern Medical University Animal Care and Use Committee in accordance with the guidelines for the ethical treatment of animals (approval no. NFYY-2021-0120).

### Statistics.

All statistical analyses of data were conducted using R software, version 3.6.1. All experiments were done at least in triplicate unless otherwise noted. Statistical significance of the difference between 2 independent groups was determined by a 2-tailed paired Student’s *t* test. One-way ANOVA with Bonferroni’s post hoc test was used for experiments with more than 2 groups. Data are expressed as mean ± SD. *P* < 0.05 was deemed statistically significant.

### Study approval.

All experiments were endorsed by the Ethics Committee of Southern Medical University and complied with the Declaration of Helsinki. All animal experiments were carried out with the approval of the Southern Medical University Animal Care and Use Committee in accordance with the guidelines for the ethical treatment of animals. All animal experiments involved ethical and humane treatment under a license from the Guangdong Provincial Bureau of Science.

### Data availability.

All data generated or analyzed during this study are included in the article and the Supporting Data Values XLS file. The microarray data reported here have been submitted to the GEO (GSE208551 and GSE236091).

## Author contributions

YM and ZH contributed to the study design and manuscript preparation. KL, FL, and YH conducted the experiments. KL, YG, DF, and PS analyzed the data. QQ, LD, JW, RC, ZF, and BL provided suggestions during development of the study design.

## Supplementary Material

Supplemental data

Supporting data values

## Version 1 11/02/2023

In-Press Preview

## Version 2 12/22/2023

Electronic publication
