# Supplementary material for: The homing of exogenous hair follicle mesenchymal stem cells into hair follicle niches
Source: JCI Insight. 2023 Dec 22;8(24):e173549. doi: 10.1172/jci.insight.173549 (PMC10807717; doi:10.1172/jci.insight.173549)
Supplement: Supplemental data [file jciinsight-8-173549-s152.pdf]

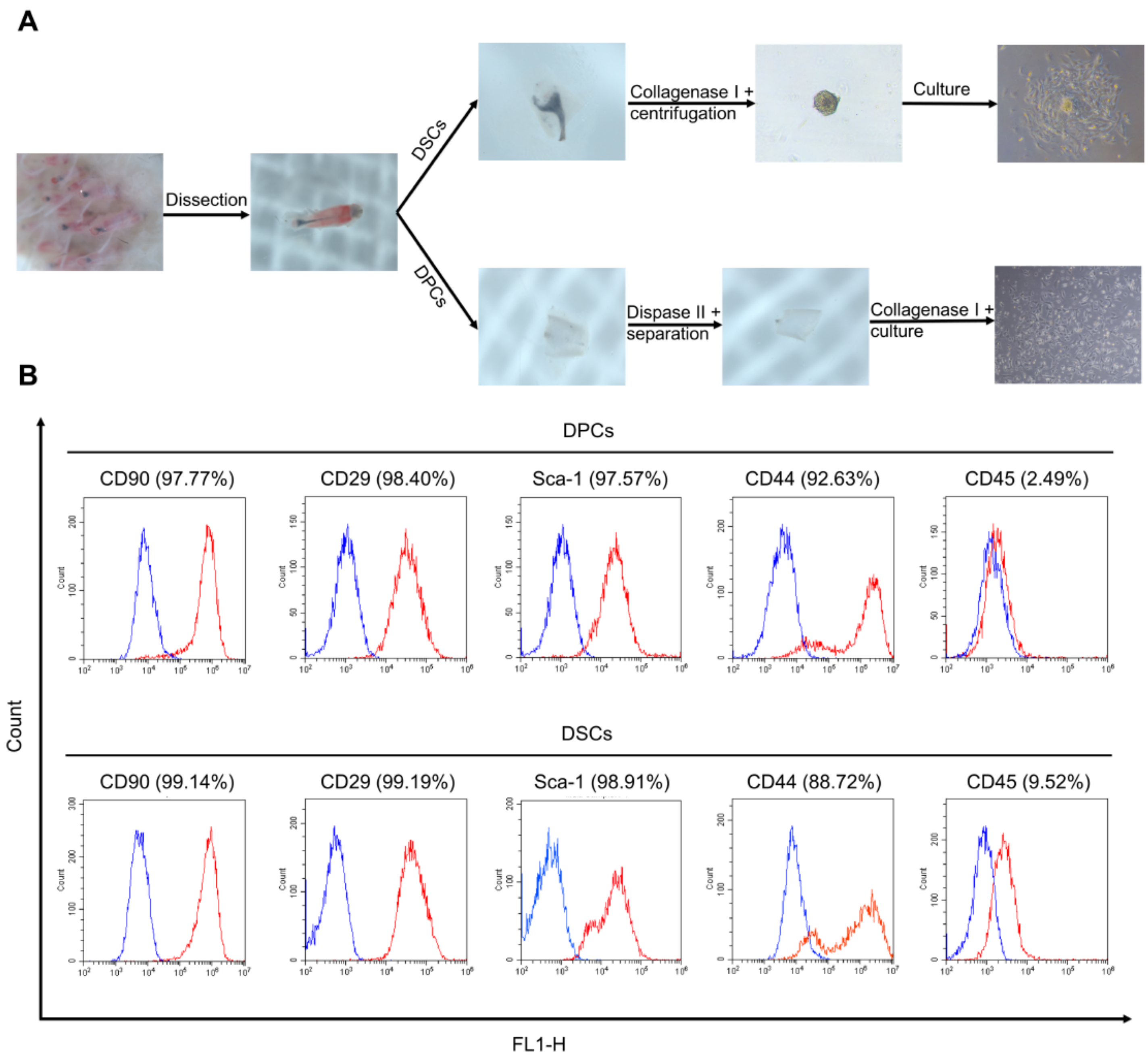

Supplemental Figure 1. Isolation and identification of MSCs markers in DPCs and DSC. (A) The procedure for isolating DPCs and DSCs is depicted. (B) Flow cytometry experiments were performed to assess the expression of MSC markers in DPCs and DSCs (representative of 3 experiments).

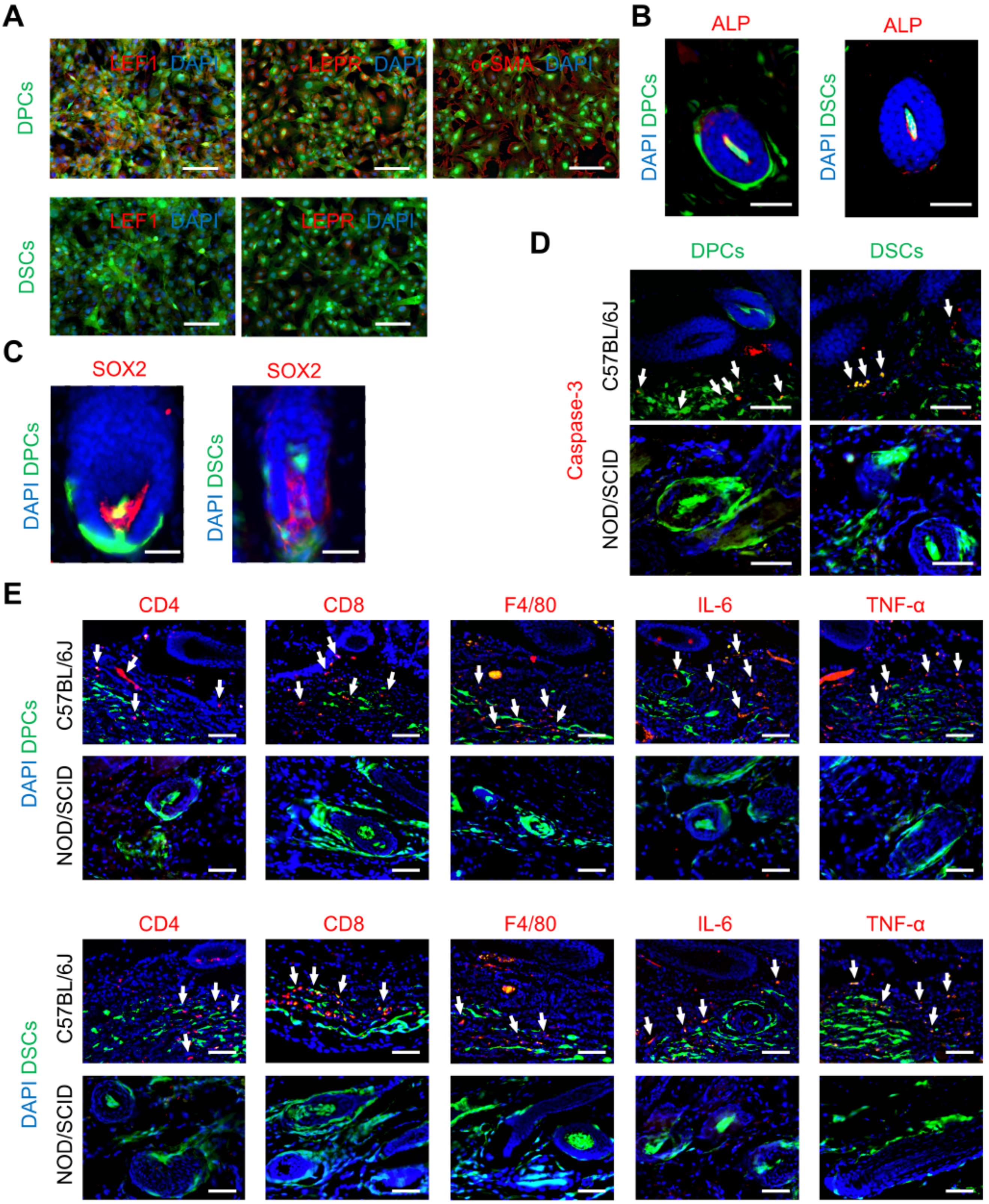

Supplemental Figure 2. Expression of follicle niche markers in homing hfMSCs and immunological rejection of the injected cells within C56BL/6 mouse. (A) Immunostaining for LEF1, LEPR and α-SMA in 3rd-passage DPCs; Immunostaining for LEF1 and LEPR in 3rd-passage DSCs (representative of 3 experiments). (B) HFs sections were harvested 2 weeks after intradermal injection of DPCs and DSCs into depilated NOD/SCID mouse skin. HFs containing exogenous DPCs (green) and DSCs (green) were immunostained for ALP (red) (representative of 3 experiments). (C) HF sections were harvested 6 months after intradermal injection of DPCs and DSCs into depilated NOD/SCID mouse skin. HFs containing exogenous DPCs (green) and DSCs (green) were immunostained for SOX2 (red) (representative of 3 experiments). (D and E) HFs sections were harvested 2 weeks after intradermal injection of DPCs and DSCs into depilated NOD/SCID mouse and C57BL/6J mouse skin. The skin sections were immunostained for Caspase-3, CD4, CD8, F4/80, IL-6 and TNF-α (red, arrow) (representative of 3 experiments). Scale bars represent 100 μm in (A); 25 μm in (C); 50 μm in (B, D and E).

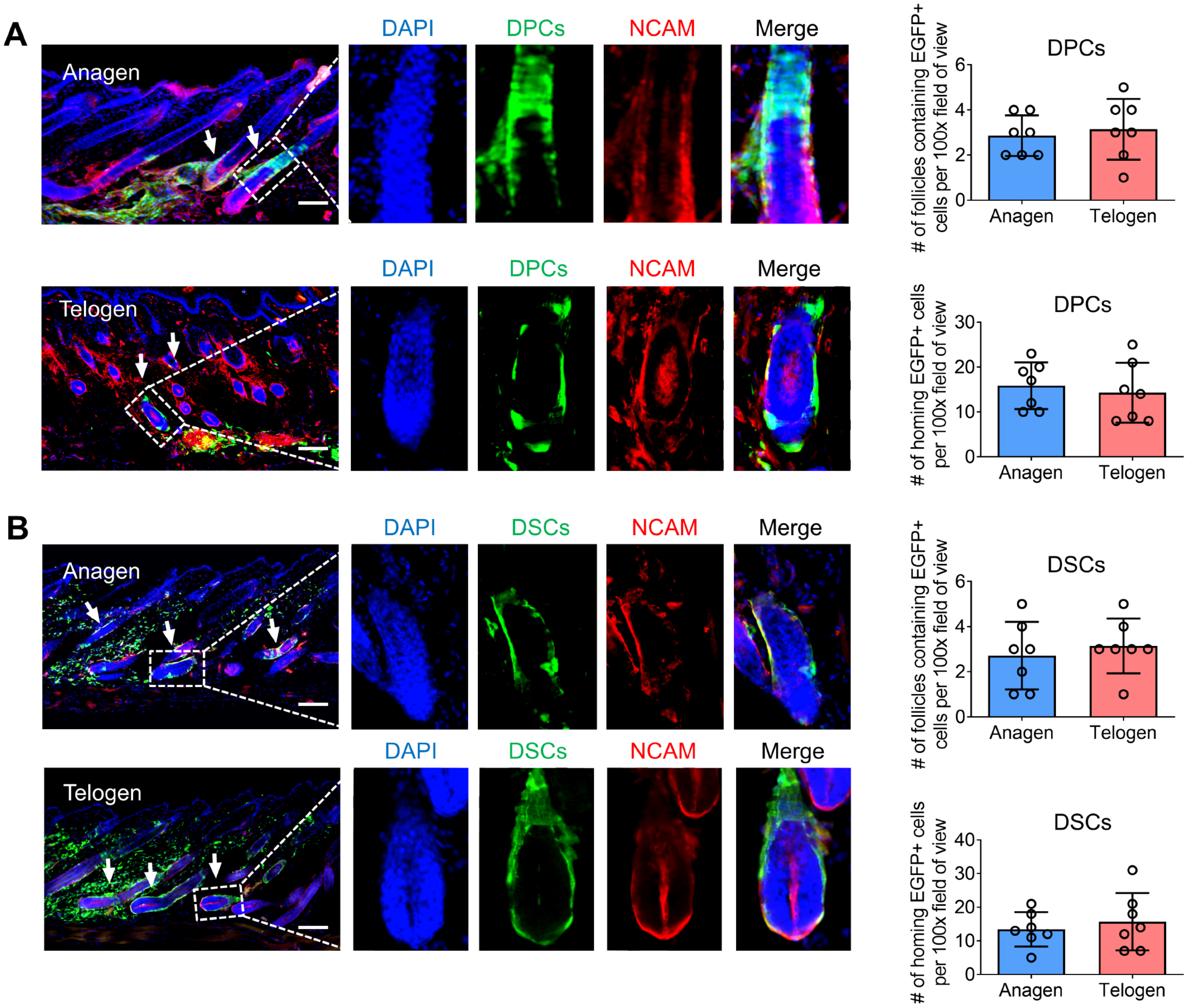

Supplemental Figure 3. HF cycle does not affect the homing of exogenous hfMSCs. (A and B) Dorsal skin sections were harvested 2 weeks after intradermal injection of DPCs and DSCs into anagen and telogen mouse skin, HFs containing exogenous DPCs (green, arrow) or DSCs (green, arrow) were immunostained for NCAM (red). Number of HFs containing EGFP+ transplanted cells and number of homing EGFP+ cells per 100× field of view (n = 7 skin sections from 4 mice per group). 2-tailed Student's t-test, mean ± SD, *P<0.05; **P<0.01; *** P<0.001. Scale bars represent 100 μm.

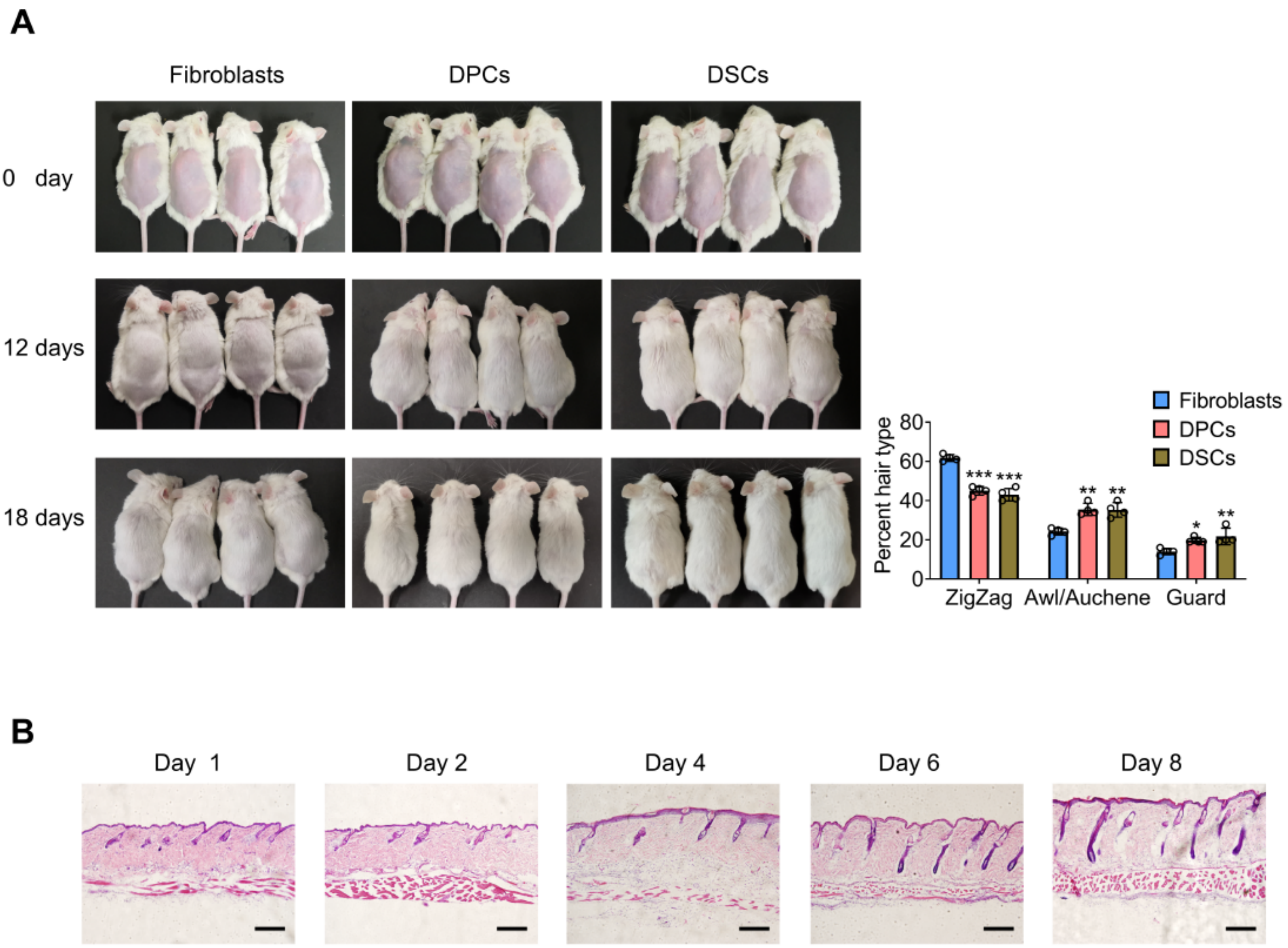

Supplemental Figure 4. Exogenous hfMSCs promote HF growth. (A) Assessment of hair growth in recipient mice after injection of DPCs, DSCs, and non-DPC, non-DSC dermal fibroblasts. Percentage of hairs of each type in mice of fibroblasts, DPCs and DSCs groups 12 days after depilation (n = 4 mice per group). 1-way ANOVA followed by Bonferroni's post hoc test, mean ± SD, *P<0.05; **P<0.01; *** P<0.001. (B) Hematoxylin-Eosin staining showed HF morphogenesis at different times after skin depilation (representative of 3 experiments). Scale bars represent 400 μm.

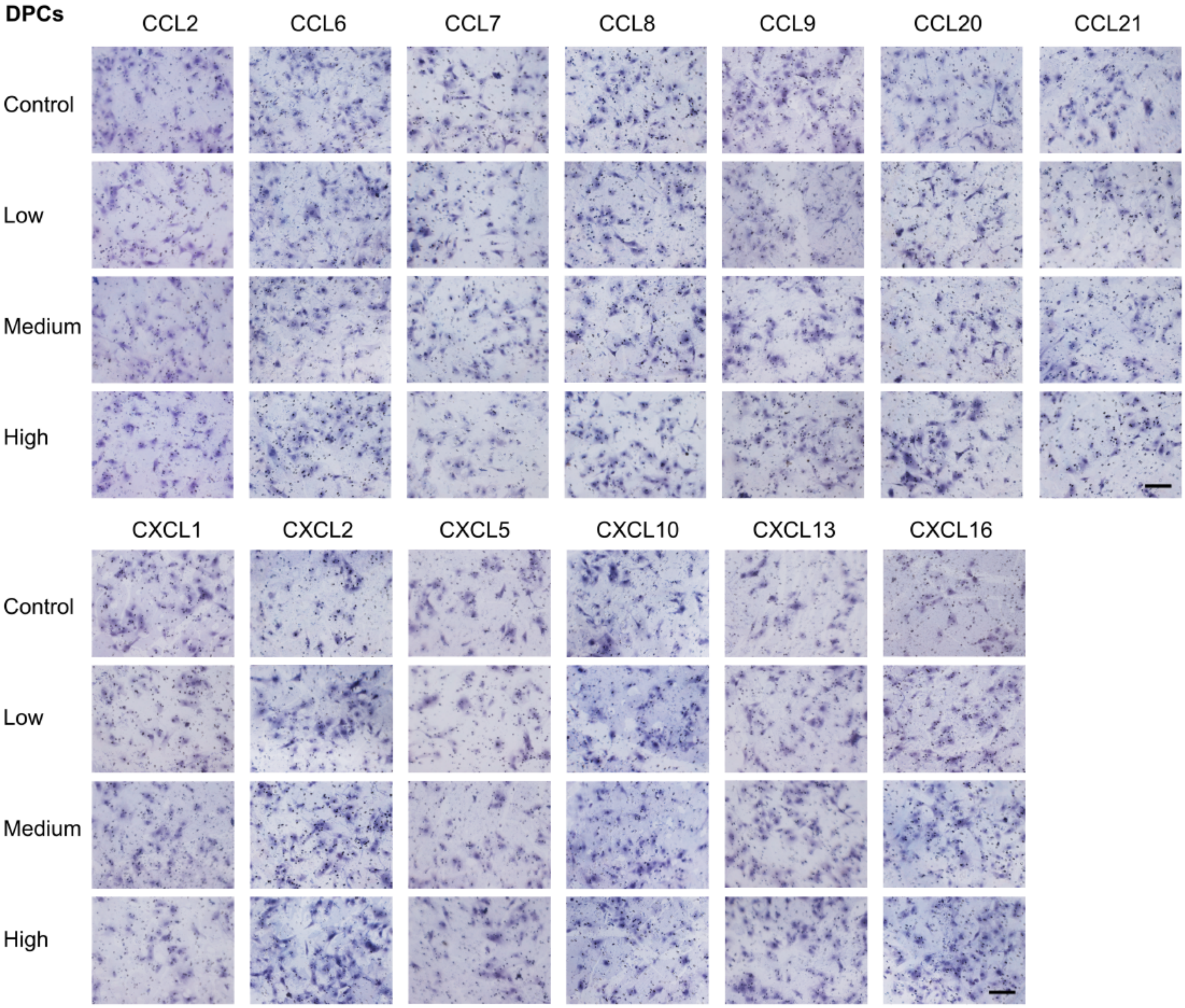

Supplemental Figure 5. Transwell chemotaxis assays showed the migratory response of DPCs to the sorted chemokines. The cells were grouped according to the concentration of chemokines (namely, Control: 0 ng/ml; Low: 5 ng/ml; Medium: 50 ng/ml; High: 500ng/ml). Scale bars represent 100 μm.

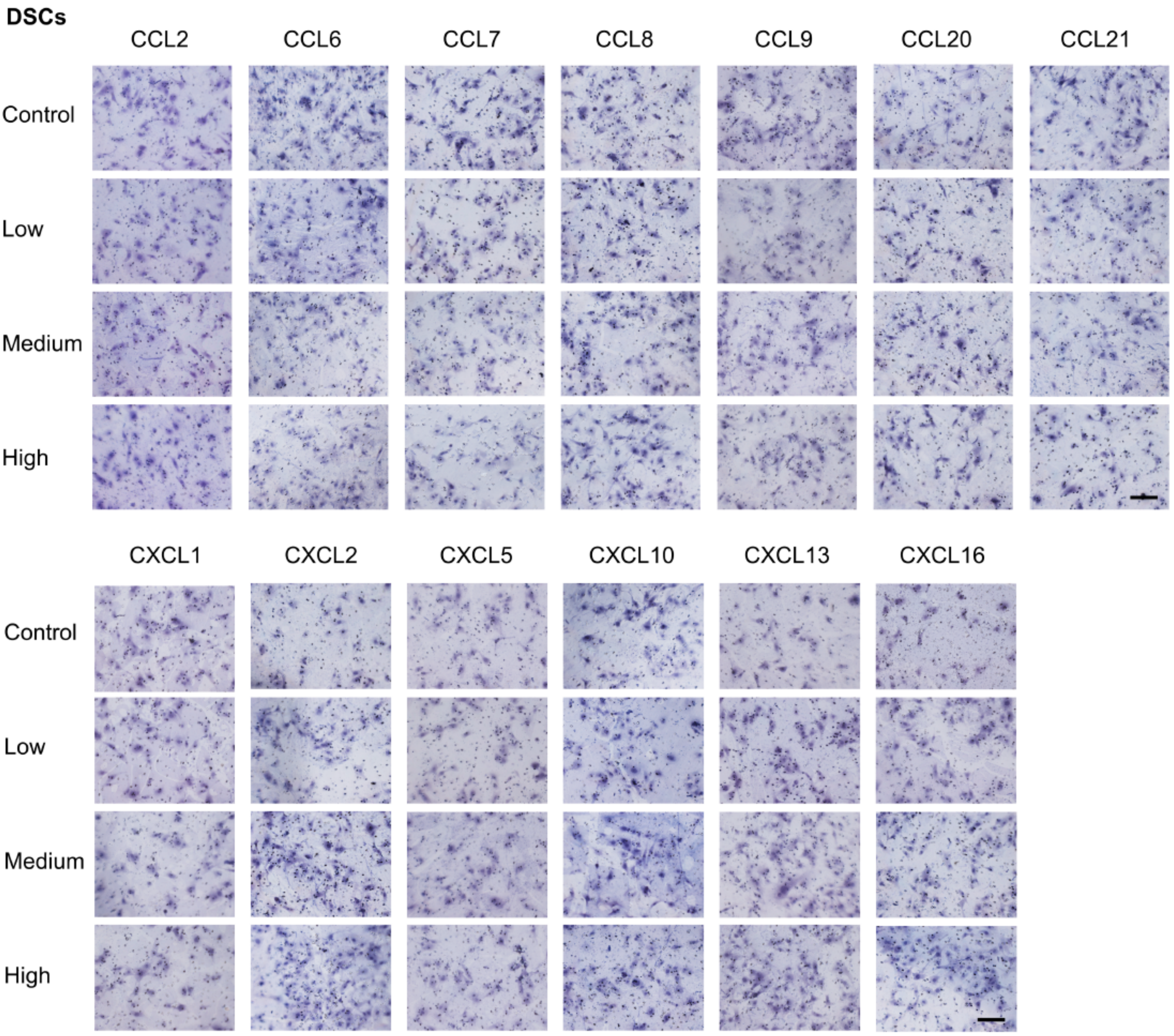

Supplemental Figure 6. Transwell chemotaxis assays showed the migratory response of DSCs to the sorted chemokines. The cells were grouped according to the concentration of chemokines (namely, Control: 0 ng/ml; Low: 5 ng/ml; Medium: 50 ng/ml; High: 500ng/ml). Scale bars represent 100 µm.

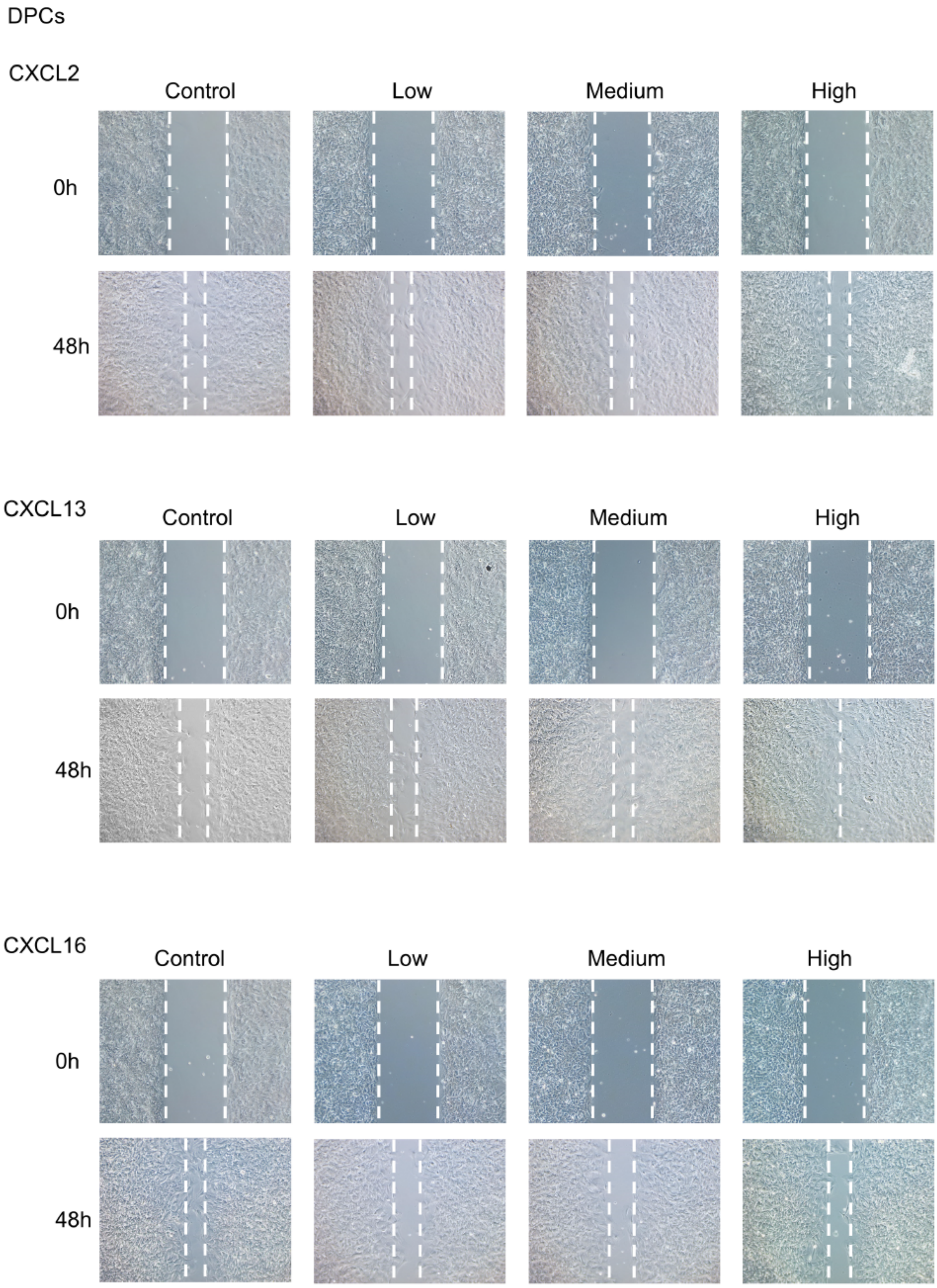

Supplemental Figure 7. Wound healing assays showed the chemotaxis effects of CXCL2, CXCL13 and CXCL16 on DPCs. The cells were grouped according to the concentration of chemokines (namely, Control: 0 ng/ml; Low: 5 ng/ml; Medium: 50 ng/ml; High: 500ng/ml).

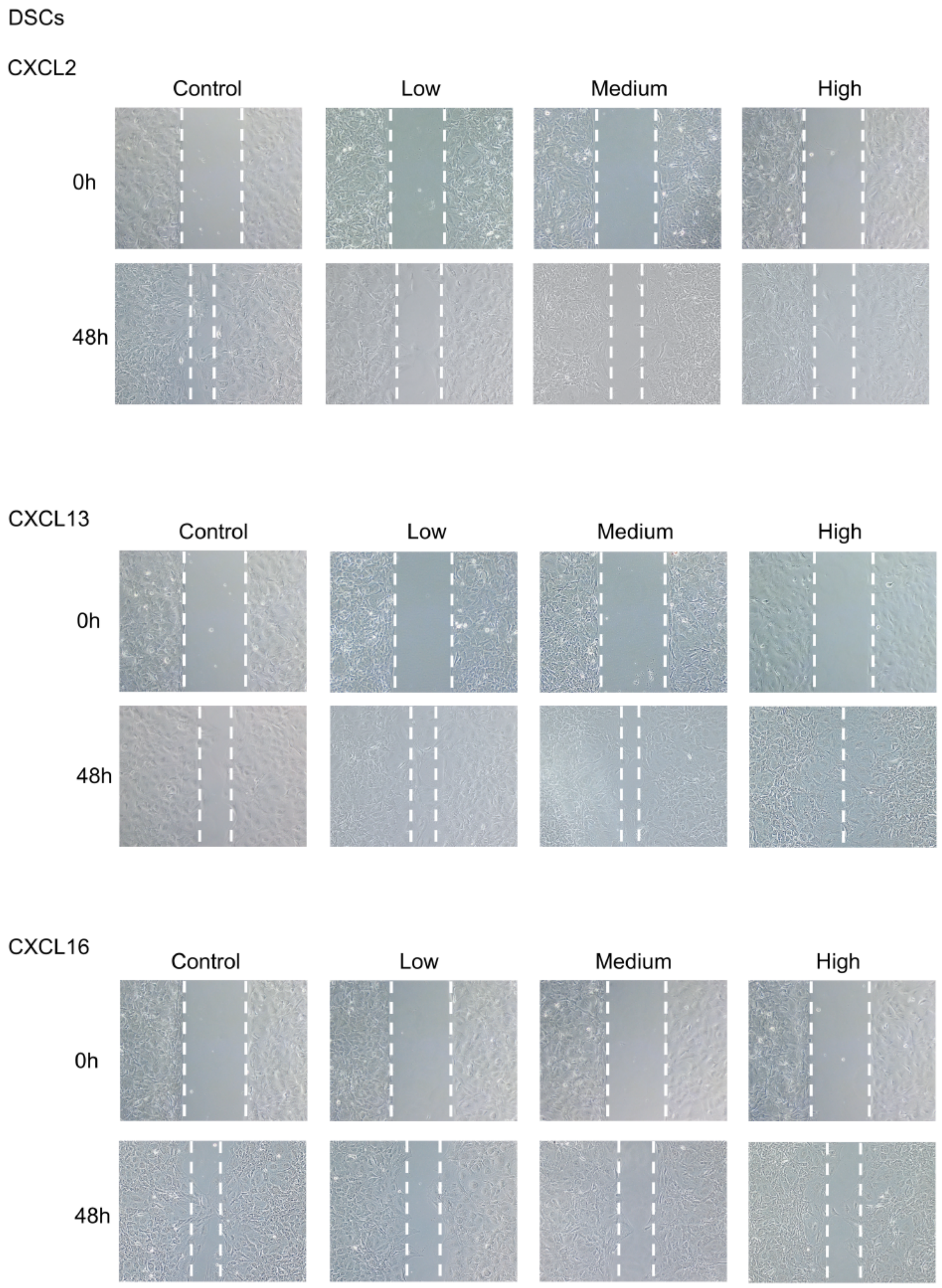

Supplemental Figure 8. Wound healing assays showed the chemotaxis effects of CXCL2, CXCL13 and CXCL16 on DSCs. The cells were grouped according to the concentration of chemokines (namely, Control: 0 ng/ml; Low: 5 ng/ml; Medium: 50 ng/ml; High: 500ng/ml).

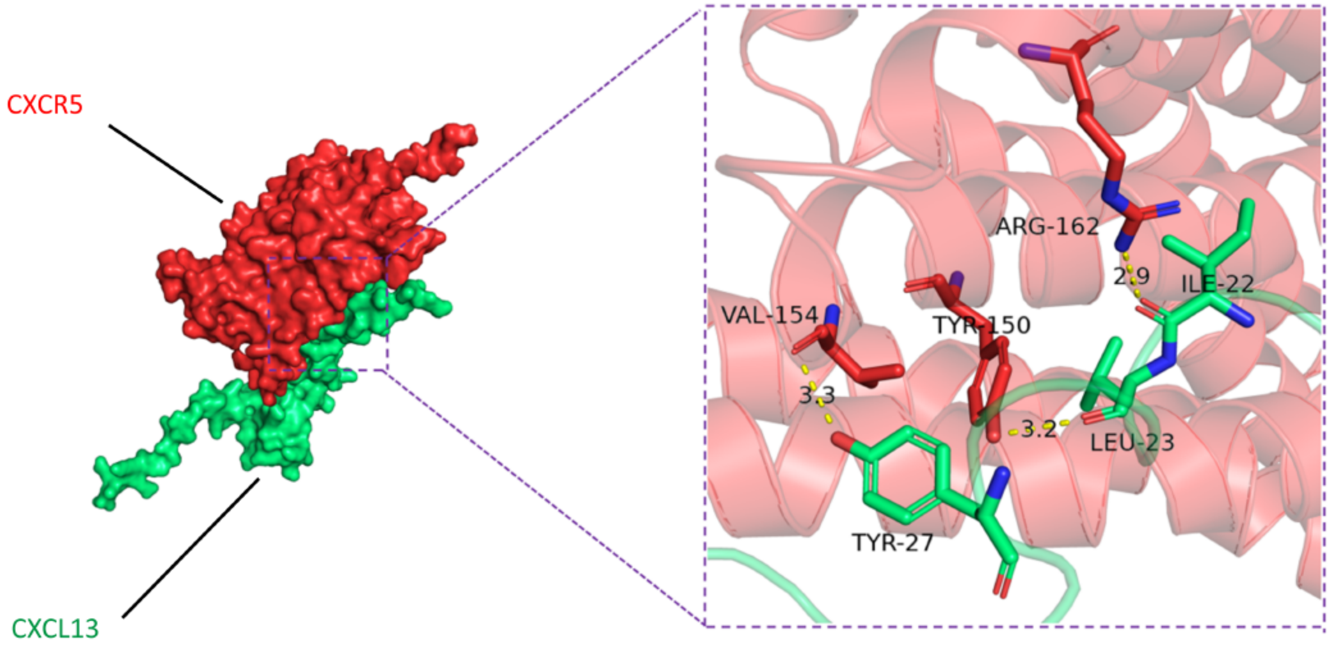

Figure S9. Molecular docking analysis of the interaction between CXCL13 and CXCR5.

Supplementary Methods and Materials

Flow cytometry analysis of cell surface marker expression

Mouse peripheral blood cells were firstly treated with red cell lysing buffer (Leagene, Beijing, China) for 10 min at 4 °C, and washed with phosphate-buffered saline (PBS from Invitrogen) twice. Cells were then suspended in PBS and stained by CD3-APC (BioLegend, 100235), CD4-PE (BioLegend, 100407) and CD8-FITC (Biorbyt, orb432810) antibodies for 30 min at 4 °C. The T lymphocytes was determined via analysing the data using BD FACSDiva™ Software.

Freshly isolated DPCs and DSCs (from adherent cultures) were characterized using flow cytometry analysis for their cell surface marker expression. Cells were trypsinized, collected, and washed with PBS twice. Cells were then suspended at a density of 10^6 /mL in PBS and stained with fluorescein isothiocyanate and phycoerythrin-conjugated monoclonal antibodies for 15 min at room temperature. The cell surface markers include CD90 (BioLegend, 105201), CD29 (BioLegend, 102201), Sca-1 (BioLegend, 160907), CD44 (BioLegend, 156007) and CD45 (BioLegend, 157213). For the control group, cells were stained with corresponding immunoglobulin isotypes.

Lentiviruses transfection and transient transfection

Lentiviruses overexpressing mouse CXCR5-shRNA and empty vector were built by Shandong Vigene Biosciences Co., Ltd. (Shandong, China). The CXCR5-shRNA sequences were shown in Supplemental Table 3. For lentiviral transfection of DPCs and DSCs cells, 100 multiplicity of infection CXCR5-shRNA or empty vector lentiviruses

were added to a well containing 5x10^4 cells, medium and 8 μg/ml polybrene. After 24 h of incubation. Empty vector lentivirus was used as a control.

CXCR5-shRNA plasmids were purchased from Shandong Vigene Biosciences Co., Ltd. (Shandong, China). DPCs/DSCs at exponential growth phase were plated into 6-well plates for 24 h at a density of 0.5 × 10^5 cells/mL, and transfected with 4 μg plasmid using Lipofectamine 2000 reagent for 24 h (Invitrogen; Carlsbad, Calif, USA) in reduced serum medium (OPTI-MEM-I; Invitrogen) according to the manufacturer's protocol.

Skin sample preparation for RNA preparation and microarray

To assess chemokine expression after NOD/SCID mice depilation, wax stripping was performed and the skin samples were collected at different time points after waxing, including 1h, 6h, 12h, 1 day, 2 days, 4 days and 6 days. To reduce the variation, we collect skin samples from three mice at each time point. After sacrificing the mice, the whole back skin was isolated and ground with liquid nitrogen. After grinding, total RNA of back skin was extracted using Trizol reagent (Invitrogen, Carlsbad, CA, USA) following the manufacturer’s recommendations. RNA quality and quantity of all samples were evaluated by using Bioanalyzer 2100 and RNA 6000 Nano LabChip Kit (Agilent, CA, USA) with RIN number > 7.5. Qualified RNAs were used for further mRNA-seq analysis and qRT-PCR.

RT-qPCR

Total RNA was polyadenylated and underwent reverse transcription using PrimeScript™ RT Master Mix (TaKaRa, Dalian, China). RT-qPCR was carried out using a SYBR®

Premix Ex TaqTM II (TaKaRa, Dalian, China) on a RocheLightCycler480 system. GAPDH was used as an endogenous control. All samples were normalized to internal controls, and fold changes were calculated through relative quantification ($2^{-\Delta\Delta CT}$). The primers used are shown in **Supplemental Table 2**.

RNA library construction and sequencing

The mRNA was isolated from approximately 6 μg (1μg per sample) of total RNA. Sequencing libraries were generated using NEBNext®UltraTM RNA Library Prep Kit for Illumina® (NEB, Ipswich, USA) following the manufacturer's instructions. Briefly, mRNA was purified from total RNA using poly-T oligo-attached magnetic beads. Then the mRNA was fragmented into small pieces and then were reverse-transcribed into cDNA to create the final cDNA library. The library preparations were sequenced on an Illumina Hiseq 2500 platform and paired-end reads were generated.

cDNA library preparation

Poly-A-containing mRNA molecules were purified using poly-T oligo-attached magnetic beads. After adding divalent cations, the mRNA was disrupted into short fragments under elevated temperature in NEB Next First Strand Synthesis Reaction Buffer (5X). First-strand cDNA was synthesized using reverse transcriptase (RNase H) and random primers and second-strand cDNA was synthesis with DNA polymerase I and RNaseH. Through exonuclease/polymerase activities, the remaining overhangs were converted into blunt ends. Following adenylation of 3' ends of DNA fragments, NEBNext Adaptor with hairpin loop structure was ligated for hybridization. Then the products were purified with AMPure XP system (Beckman Coulter, Beverly, USA) to select cDNA fragments of

200-250 bp in length. The adaptor-ligated cDNA was then purified and enriched with PCR using Phusion High-Fidelity DNA polymerase, Universal PCR primers and Index (X) Primer. Finally, the cDNA libraries were validated on the Agilent Bioanalyzer 2100 system.

Immunofluorescent staining

Skin tissues were collected and fixed in 4% paraformaldehyde in phosphate-buffered saline (PBS), dehydrated, embedded in OCT compound, and sectioned at 8 to 10 μm. All sections were permeabilized in 0.5% Triton X-100 in PBS and blocked for 1 h with 0.5% BSA in PBS. Next, sections were washed in PBS, and incubated with primary antibodies at 4°C overnight. Antibodies and dilutions used were: NCAM (Abcam, ab220360), α-SMA (Abcam, ab32575), ALP (Affinity, AF2910-SP), SOX2 (Affinity, AF2018-SP), LEF1 (Abcam, ab137872), LEPR (Invitrogen, MA5-32685), CD4 (Abcam, ab183685), CD8 (Abcam, ab217344), F4/80 (Abcam, ab300421), IL-6 (Abcam, ab290735), TNF-α (Abcam, ab1793), β-catenin (Abcam, ab32572), Ki67 (Abcam, ab16667), Caspase-3 (Abcam, ab13847), CXCL16 (Affinity, DF13312), CXCL2 (R&D, AF-452-NA), CXCL13 (GeneTex, GTX108471), CXCR5 (Abcam, ab133706). After thorough rinsing with PBS, samples were incubated with Alexa Fluor-488 conjugated anti-mouse secondary antibody (Abcam, ab150117) or Alexa Fluor-594 conjugated anti-rabbit secondary antibody (Abcam, ab150080) for 1 h at room temperature. Nuclei were stained using 4 diamidino-2-phenylindole (DAPI).

Imaging was performed using Olympus BX63 microscope (Tokyo, Japan) and analyzed

using cellSens software (Olympus, Tokyo, Japan). Immunostaining intensity was assessed by quantitative immunohistomorphometry using ImageJ software (National Institutes of Health, Bethesda, MD), as described previously (1).

Transwell chemotaxis assays

Cell migration was determined using 24-well transwell chambers with 8 μm pore size polycarbonate membranes (Corning Star; Cambridge, Mass, USA). 1×10^5 cells were seeded on the top side of the membrane. The bottom chamber was prepared using 2% FBS with 0 ng/ml, 5 ng/ml, 50 ng/ml and 500ng/ml chemokines (CCL2, CCL6, CCL8, CCL9, CCL20, CCL21a, CCL21b, CXCL1, CXCL2, CXCL5, CXCL10, CXCL13 and CXCL16, purchased from PeproTech). Cells were allowed to migrate through the porous membrane for 24 h at 37°C. The cells that stuck to the lower surface of the membrane were treated with a fixation/staining solution (ethanol, hematoxylin) for visualization. The cells were counted under a microscope in 5 randomly selected fields (original magnification, ×200). At least 4 chambers from 3 different experiments were analyzed.

Wound healing assay

Cell suspensions were prepared and seeded with Culture-Insert (Ibidi 80206, American) into each plate. Then, 70ul of cell suspension was placed into each well. The inserts were incubated at 37℃ in 5% CO2, and were removed with sterile tweezers after 24h. The used wells were filled with DMEM and after treatment with 0 ng/ml, 5 ng/ml, 50 ng/ml and 500ng/ml chemokines (CCL2, CCL6, CCL8, CCL9, CCL20, CCL21a, CCL21b, CXCL1, CXCL2, CXCL5, CXCL10, CXCL13 and CXCL16, purchased from

PeproTech), the wound area was imaged with a reverse phase-contrast microscope (IX61 FL, Olympus, Japan) immediately, after 48h. The mean migration distance (μm) was calculated by subtracting the length after 48 h from that at 0 h. The result was expressed as a migration index, ie, the distance migrated by treated cells compared with the distance migrated by control cells.

Supplemental Table 1. The receptors of chemokines up-regulated after skin depilation

Chemokines	Chemokines receptors
CCL2	CCR2 CCR11
CCL6	CCR1 CCR2 CCR3
CCL7	CCR1 CCR2 CCR3 CCR4
CCL8	CCR5 CCR8 CCR1 CCR2 CCR11
CCL9	CCR1
CCL20	CCR6
CCL21a	CCR7 CCR11
CCL21b	CCR7 CCR11
CXCL1	CXCR1 CXCR2 CXCR6
CXCL2	CXCR2
CXCL5	CXCR1 CXCR2
CXCL10	CXCR3
CXCL13	CXCR5 CXCR3
CXCL16	CXCR6

Supplemental Table 2. RT-PCR primer sequences for chemokines and chemokine receptors

Gene name	Forward primer	Reverse primer
CCL2	TTAAAAACCTGGATCGGAACCAA	GCATTAGCTTCAGATTTACGGGT
CCL6	GCTGGCCTCATACAAGAAATGG	GCTTAGGCACCTCTGAACTCTC
CCL7	GCTGCTTTCAGCATCCAAGTG	CCAGGGACACCGACTACTG
CCL8	TCTACGCAGTGCTTCTTTGCC	AAGGGGGATCTTCAGCTTTAGTA
CCL9	CCCTCTCCTTCCTCATTCTTACA	AGTCTTGAAAGCCCATGTGAAA
CCL20	GCCTCTCGTACATACAGACGC	CCAGTTCTGCTTTGGATCAGC
CCL21a	GTGATGGAGGGGGTCAGGA	GGGATGGGACAGCCTAAACT
CCL21b	TCCCTACAGTATTGTCCGAGGC	ATCAGGTTCTGCACCCAGCCTT
CXCL1	CTGGGATTCACCTCAAGAACATC	CAGGGTCAAGGCAAGCCTC
CXCL2	CCAACCACCAGGCTACAGG	GCGTCACACTCAAGCTCTG
CXCL5	GTTCCATCTCGCCATTCATGC	GCGGCTATGACTGAGGAAGG
CXCL10	CCAAGTGCTGCCGTCATTTTC	GGCTCGCAGGGATGATTTCAA
CXCL13	GGCCACGGTATTCTGGAAGC	GGGCGTAACTTGAATCCGATCTA
CXCL16	CCTTGTCTCTTGCGTTCTTCC	TCCAAAGTACCCTGCGGTATC
CCR1	CTCATGCAGCATAGGAGGCTT	ACATGGCATCACCAAAAATCCA
CCR2	TGTGATTGACAAGCACTTAGACC	TGGAGAGATACCTTCGGAACTT
CCR3	TCAACTTGGCAATTTCTGACCT	CAGCATGGACGATAGCCAGG
CCR4	TGCACCAAGGAAGGTATCAAGG	GTACACGTCCGTCATGGACTT
CCR5	ATGGATTTTCAAGGGTCAGTTCC	CTGAGCCGCAATTTGTTTCAC
CCR6	ATGCGGTCAACTTTAACTGTGG	CCCGGAAAGATTTGGTTGCCT
CCR7	GCCCAGATGGTTTTTGGGTTC	GCAAGGTACGGATGATAATGAGG
CCR8	ACGTCACGATGACCGACTACT	CCCAGCACAAACAAGACGC
CCR11	CGGATGGATTCTAGGCAAAATGA	GCCCAATATCTGTCAATGCTGA
CXCR1	TGCTGGTTATCTTATACAGGCGA	CAGCCCTTCAATTTGGAGACA
CXCR2	TGTCTGGGCTGCATCTAAAGT	AGGTAACCTCCTTCACGTATGAG
CXCR3	AAAACAGCACCTCTCCCTA	TCTGAACTTCACTCCCACA
CXCR5	GTAGCCGACCTTCTCTTAGTCT	GTGCAGAGCGATCACAGTTTT
CXCR6	GAGTCAGCTCTGTACGATGGG	TCCTTGAACTTTAGGAAGCGTTT
GAPDH	AGGTCGGTGTGAACGGATTTG	TGTAGACCATGTAGTTGAGGTCA

Supplemental Table 3. The sequences of CXCR5-shRNA

shRNA	Sequences
CXCR5-shRNA1	GCTCCATCACATACAATATGGTTCAAGAGACCATATTGTATGTGATGGAGCTTTTTT
CXCR5-shRNA2	CCTCATAACAACGACTCCTTATTCAAGAGATAAGGAGTCGTTGTTATGAGGTTTTTT
CXCR5-shRNA3	GCTTGTGATGGGATGGTGTTATTCAAGAGATAACACCATCCCATCACAAGCTTTTTT
CXCR5-shRNA4	TGGCACACTGCTGTCTCAATCTTCAAGAGAGATTGAGACAGCAGTGTGCCATTTTTT

Supplemental Table 4. Up-regulated genes in high-passage DPCs and DSCs.

Gene ID	Gene Name	log2FC(DPCs_high vs DPCs_low)	log2FC(DSCs_high vs DSCs_low)
ENSMUSG00000110869	AC122901.1	9.072347521	9.613592164
ENSMUSG00000019772	Vip	2.208695182	8.600381148
ENSMUSG00000087516	Tbx3os1	3.535496895	7.371829643
ENSMUSG00000098415	Mir6928	2.744090896	7.045977601
ENSMUSG00000114194	AC154552.9	2.73078154	6.158805486
ENSMUSG00000031722	Hp	5.165017682	5.986040861
ENSMUSG00000098279	Mir6908	6.671171593	5.68306871
ENSMUSG00000048424	Ranbp3l	5.392317927	5.676502998
ENSMUSG00000098926	Mir6904	2.140187175	5.625558816
ENSMUSG00000081497	Gm15560	2.521072165	5.565870615
ENSMUSG00000030043	Tacr1	2.723952529	5.191137485
ENSMUSG00000095836	Gm21960	2.028126208	5.084591493
ENSMUSG00000081099	Gm5270	6.654196023	4.939385627
ENSMUSG00000042751	Nmnat2	2.154813645	4.913448608
ENSMUSG00000035948	Acss3	2.028508878	4.806873676
ENSMUSG00000099063	Mir7031	2.349060024	4.764807115
ENSMUSG00000098701	Mir6914	4.492794297	4.730843289
ENSMUSG00000102798	Gm37271	2.045020747	4.70261612
ENSMUSG00000087247	Fam150a	2.442907998	4.691292574
ENSMUSG00000021974	Fgf9	2.867020319	4.689547487
ENSMUSG00000046613	Vwa5b2	4.70532391	4.642736516
ENSMUSG00000020684	Rasl10b	4.034137593	4.637690061
ENSMUSG00000043613	Mmp3	5.450211473	4.547962245
ENSMUSG00000014813	Stc1	5.60931368	4.469539667
ENSMUSG00000054889	Dsp	4.476684634	4.36958062
ENSMUSG00000022219	Cideb	4.305862148	4.358134432
ENSMUSG00000036196	Slc26a8	4.26753474	4.226077379
ENSMUSG00000030351	Tspan11	2.053025445	4.20635228
ENSMUSG00000107928	Gm45140	4.313992495	4.170645693

ENSMUSG00000105021	Gm8234	2.422217628	4.164486008
ENSMUSG00000093206	Gm22413	5.044335389	4.135582934
ENSMUSG00000103574	Gm37116	2.485945457	4.132159194
ENSMUSG00000036862	Dchs1	5.732910907	4.07640224
ENSMUSG00000049313	Sorl1	4.710290607	3.997387216
ENSMUSG00000068859	Sp9	4.59855426	3.977511789
ENSMUSG00000047013	Fbxo41	3.242472533	3.968829493
ENSMUSG00000031548	Sfrp1	6.751982106	3.932350065
ENSMUSG00000056553	Ptprn2	4.303397882	3.927058751
ENSMUSG00000022156	Gzme	3.128313221	3.894787221
ENSMUSG00000017740	Slc12a5	4.455095993	3.861727354
ENSMUSG00000103749	Pcdhgb5	3.30585398	3.832139896
ENSMUSG00000093107	Mir1839	3.27521605	3.804109997
ENSMUSG00000036585	Fgf1	4.376460651	3.766870516
ENSMUSG00000030222	Rerg	4.372638991	3.728008143
ENSMUSG00000024697	Gna14	3.639865187	3.711128806
ENSMUSG00000055639	Dach1	2.137053746	3.708965921
ENSMUSG00000033491	Prss35	7.138809907	3.640017005
ENSMUSG00000065561	Mir369	6.134382438	3.631646127
ENSMUSG00000011154	Cfap161	4.244618126	3.608617214
ENSMUSG00000019359	Gdpd2	5.557609596	3.564581521
ENSMUSG00000085228	Gm14376	2.399951109	3.561604666
ENSMUSG00000000359	Rem1	5.185398571	3.524073049
ENSMUSG00000051022	Hs3st1	3.531400302	3.490937328
ENSMUSG00000032648	Pygm	2.803160399	3.481723636
ENSMUSG00000026768	Itga8	2.352726754	3.465226647
ENSMUSG00000014773	Dll1	2.430293862	3.463570413
ENSMUSG00000078439	Smim24	2.039303334	3.448549517
ENSMUSG00000067279	Ppp1r3c	2.969109955	3.438097959
ENSMUSG00000100959	Gm7654	2.629470007	3.436799028
ENSMUSG00000106927	Gm43598	2.722993414	3.407745396
ENSMUSG00000020871	Dlx4	3.070581318	3.404405884
ENSMUSG00000105895	Gm42829	5.139561797	3.388967757
ENSMUSG00000059149	Mfsd4a	3.055987087	3.387958515
ENSMUSG00000097140	Gm26779	2.496111434	3.371182529
ENSMUSG00000042961	Egflam	3.481857524	3.36108704
ENSMUSG00000028864	Hgf	3.828323611	3.354011331
ENSMUSG00000099032	Tcf24	2.061436994	3.333260238
ENSMUSG00000027071	P2rx3	2.060541093	3.326754199
ENSMUSG00000025892	Gria4	3.464217289	3.306425439

ENSMUSG00000099517	Hist1h3g	2.031521956	3.251231298
ENSMUSG00000044164	Rnf182	4.394040217	3.249333483
ENSMUSG00000030137	Tuba8	3.575379872	3.242635878
ENSMUSG00000067158	Col4a4	2.842429987	3.20434152
ENSMUSG00000040380	Cbln3	2.32914909	3.19858863
ENSMUSG00000091163	Gm3962	3.310937504	3.190747227
ENSMUSG00000069227	Gprin1	3.723502422	3.174823533
ENSMUSG00000090955	Gm17097	3.622216703	3.151717951
ENSMUSG00000048834	Vstm2a	2.630992301	3.14633541
ENSMUSG00000103703	Gm42568	3.789840904	3.132126116
ENSMUSG00000044317	Gpr4	3.361080212	3.104229953
ENSMUSG00000040808	S100g	2.980591132	3.089895841
ENSMUSG00000096243	Gm24265	3.778204896	3.082769101
ENSMUSG00000082718	Gm14928	2.95870752	3.072225807
ENSMUSG00000027408	Cpxm1	4.696662039	3.014267288
ENSMUSG00000015829	Tnr	5.996872025	2.985021948
ENSMUSG00000036928	Stag3	6.119904281	2.979370145
ENSMUSG00000106580	Mir7231	5.871666054	2.976570055
ENSMUSG00000055653	Gpc3	4.335862062	2.975607571
ENSMUSG00000104563	Gm43041	2.534801913	2.961543627
ENSMUSG00000024131	Slc3a1	3.326616372	2.961196704
ENSMUSG00000047330	Kcne4	2.338593569	2.954697042
ENSMUSG00000028354	Fmn2	3.755095975	2.923393688
ENSMUSG00000102516	Gm38340	2.508723834	2.920319024
ENSMUSG00000078868	Gm14412	2.617253971	2.920027707
ENSMUSG00000077431	Gm22591	8.844576278	2.874469118
ENSMUSG00000108108	Gm44270	3.616111902	2.874291525
ENSMUSG00000090397	Gm17096	3.732495298	2.858368539
ENSMUSG00000049565	Aknad1	2.234141698	2.843299593
ENSMUSG00000078963	Hsbp1l1	2.136947639	2.839336598
ENSMUSG00000097086	Gm7672	2.904456111	2.837178418
ENSMUSG00000044544	4921513I03Rik	2.297645873	2.837036441
ENSMUSG00000107877	Gm43951	3.1283616	2.834622477
ENSMUSG00000065016	Snora3	3.814118624	2.822774547
ENSMUSG00000095097	Gm23658	2.458283303	2.820616735
ENSMUSG00000044296	Zfp879	2.326503489	2.804539779
ENSMUSG00000048368	Omd	2.173399421	2.778319678
ENSMUSG00000058656	Samd12	2.203452352	2.775949118
ENSMUSG00000085201	Nr6a1os	4.085593465	2.767455845
ENSMUSG00000095649	Gm8979	2.145141741	2.759773134

ENSMUSG00000093989	Rnasek	3.104522954	2.759512744
ENSMUSG00000098904	Mir6998	3.00659733	2.754385979
ENSMUSG00000026840	Lamc3	4.193732366	2.751463923
ENSMUSG00000032719	Sbspon	2.756198875	2.73859547
ENSMUSG00000039716	Dock3	3.268453137	2.733842687
ENSMUSG00000112441	AC122860.2	2.080639286	2.733575347
ENSMUSG00000040121	Rep15	2.889046732	2.721804187
ENSMUSG00000000320	Alox12	2.712127485	2.721777739
ENSMUSG00000032595	Cdhr4	5.242781184	2.718267653
ENSMUSG00000039157	Fam102a	3.622062162	2.685324434
ENSMUSG00000025422	Agap2	4.908412791	2.67331768
ENSMUSG00000052353	Cemip	4.155203013	2.665781143
ENSMUSG00000107468	5730507A11Rik	2.634403497	2.664992281
ENSMUSG00000101628	Gm28177	2.416845104	2.656059336
ENSMUSG00000057967	Fgf18	4.344086032	2.650724002
ENSMUSG00000092644	Gm23564	2.385179004	2.648223305
ENSMUSG00000020077	Srgn	2.759061524	2.647030116
ENSMUSG00000073077	Gm7173	4.144094068	2.646263569
ENSMUSG00000055271	9330161L09Rik	4.982356873	2.641996256
ENSMUSG00000105107	Gm43412	2.517523093	2.63804247
ENSMUSG00000020000	Moxd1	3.152335642	2.623671876
ENSMUSG00000022037	Clu	2.988335233	2.623243571
ENSMUSG00000020639	Pfn4	3.133350946	2.621753531
ENSMUSG00000030321	Efcab12	2.145629543	2.619177641
ENSMUSG00000102719	Gm37760	2.176606635	2.610844472
ENSMUSG00000029419	Gm996	6.034025276	2.599113481
ENSMUSG00000038011	Dnah10	4.653157409	2.574274091
ENSMUSG00000105954	Gm42793	3.001525896	2.571344267
ENSMUSG00000020333	Acsl6	3.168534238	2.561814336
ENSMUSG00000085042	Abhd11os	2.587248272	2.558675761
ENSMUSG00000107451	Gm44421	5.643753526	2.547430023
ENSMUSG00000052415	Tchh	4.01960102	2.547287167
ENSMUSG00000078921	Tgtp2	2.096767038	2.52799872
ENSMUSG00000039375	Wdr17	3.927412547	2.511318673
ENSMUSG00000025272	Tro	3.296856851	2.504174321
ENSMUSG00000078907	Fam186b	2.614047138	2.498920245
ENSMUSG00000074627	Mroh8	2.636787682	2.493273143
ENSMUSG00000006344	Ggt5	4.248183828	2.48026088
ENSMUSG00000054679	Srsf12	3.200358378	2.475915757
ENSMUSG00000079711	Smok4a	2.655267684	2.469065619

ENSMUSG00000074093	Svip	3.61507827	2.464763532
ENSMUSG00000079017	Ifi27l2a	4.241533275	2.447795902
ENSMUSG00000021390	Ogn	3.789686853	2.446960007
ENSMUSG00000096847	Tmem151b	2.895070634	2.446478607
ENSMUSG00000024827	Gldc	2.449226683	2.440451179
ENSMUSG00000029121	Crmp1	2.384481071	2.438039193
ENSMUSG00000093720	Gm20635	3.17339006	2.437357196
ENSMUSG00000022231	Sema5a	2.346988504	2.412856057
ENSMUSG00000085936	2610307P16Rik	2.21936111	2.412447544
ENSMUSG00000097072	Foxl2os	7.02489229	2.368781375
ENSMUSG00000051243	Islr2	2.274072095	2.366993813
ENSMUSG00000039021	Ttc16	3.743717793	2.363284805
ENSMUSG00000003279	Dlgap1	2.920545462	2.363274638
ENSMUSG00000102748	Pcdhgb2	3.838512389	2.356876818
ENSMUSG00000038602	Slc35f1	2.527302997	2.356051558
ENSMUSG00000066755	Tnfsf18	2.446773833	2.355725751
ENSMUSG00000074736	Syndig1	2.917592229	2.352406645
ENSMUSG00000006542	Prkag3	3.70416971	2.349951716
ENSMUSG00000104724	Gm43162	3.962406261	2.349485047
ENSMUSG00000050556	Kcnb1	7.988179471	2.337651829
ENSMUSG00000091476	Gm16432	8.113921783	2.328498673
ENSMUSG00000079489	C030013D06Rik	2.700889433	2.31981175
ENSMUSG00000030680	Pagr1a	2.686290098	2.319608334
ENSMUSG00000107884	Gm44144	2.074227914	2.319416714
ENSMUSG00000086741	Gm15816	2.429285164	2.317583076
ENSMUSG00000039481	Nrtn	3.421948921	2.306379404
ENSMUSG00000041986	Elmod1	2.786267595	2.296003202
ENSMUSG00000111063	AC125374.1	2.782221239	2.292033014
ENSMUSG00000015599	Ttbk1	2.479671656	2.28544452
ENSMUSG00000042788	Fam166b	2.720986686	2.260647485
ENSMUSG00000042763	Maneal	4.309036536	2.242442296
ENSMUSG00000047604	Frat2	3.702212817	2.238559446
ENSMUSG00000029651	Mtus2	3.853417756	2.237150529
ENSMUSG00000021388	Aspn	2.624390002	2.235777653
ENSMUSG00000056656	Apol8	2.202756524	2.232099401
ENSMUSG00000026051	1500015O10Rik	4.179989593	2.226741843
ENSMUSG00000112922	AC158605.3	2.938426964	2.222301967
ENSMUSG00000079465	Col4a3	3.272413097	2.222072516
ENSMUSG00000029304	Spp1	2.401929024	2.219056982
ENSMUSG00000039683	Sdk1	2.288914402	2.21227944

ENSMUSG00000047139	Cd24a	2.551694926	2.20681518
ENSMUSG00000064357	mt-Atp6	2.77685889	2.200611293
ENSMUSG00000078922	Tgtp1	2.219251381	2.196871094
ENSMUSG00000096929	A330023F24Rik	2.638678038	2.195121019
ENSMUSG00000085982	9530051G07Rik	2.355556288	2.184854454
ENSMUSG00000076240	Mir702	5.608801155	2.181903514
ENSMUSG00000049357	4933408B17Rik	2.116249047	2.17950029
ENSMUSG00000000142	Axin2	6.162187013	2.179244698
ENSMUSG00000053279	Aldh1a1	2.913285418	2.177812536
ENSMUSG00000016624	Phf21b	3.765071427	2.177351157
ENSMUSG00000031893	Tsnaxip1	2.693696924	2.177123023
ENSMUSG00000111292	AC159308.4	3.386971269	2.176222147
ENSMUSG00000092192	Dyx1c1	4.681477944	2.17257074
ENSMUSG00000084440	Gm26254	2.625121799	2.161453356
ENSMUSG00000053030	Spink2	2.958029214	2.15150661
ENSMUSG00000027171	Prrg4	2.325515938	2.149005038
ENSMUSG00000104140	Gm37140	2.639253739	2.131795249
ENSMUSG00000040473	Cfap69	2.855749053	2.120628802
ENSMUSG00000104765	Gm43058	2.963705254	2.120426083
ENSMUSG00000060445	Sycp2	2.450665343	2.119125765
ENSMUSG00000044349	Snhg11	3.15614846	2.110622036
ENSMUSG00000109157	Gm44829	3.227927551	2.109300863
ENSMUSG00000106867	Gm43800	3.276599255	2.102278983
ENSMUSG00000072966	Gprasp2	3.739688471	2.098310651
ENSMUSG00000031576	Kcnu1	2.886252153	2.090028275
ENSMUSG00000074650	Gm10735	4.809411909	2.080845824
ENSMUSG00000023927	Satb1	3.403549905	2.078370403
ENSMUSG00000048484	Gm7461	2.176647335	2.076685389
ENSMUSG00000097675	1700101I11Rik	2.489213798	2.066627385
ENSMUSG00000020062	Slc5a8	4.501668397	2.064848824
ENSMUSG00000078838	Gm17382	3.440236664	2.054914587
ENSMUSG00000049092	Gpr137c	5.236934604	2.05386379
ENSMUSG00000031538	Plat	4.349012518	2.046710668
ENSMUSG00000023328	Ache	2.754196989	2.045612179
ENSMUSG00000032278	Paqr5	2.929535912	2.043673402
ENSMUSG00000101365	Gm19325	2.487175954	2.025768233
ENSMUSG00000110637	Gm7807	2.818854957	2.009798986
ENSMUSG00000093507	Gm20627	2.175442977	2.008209385
ENSMUSG00000110289	4930412F12Rik	2.088867629	2.00320732
ENSMUSG00000112000	AC153501.1	3.029272083	2.002953535

Supplemental Table 5. Down-regulated genes in high-passage DPCs and DSCs.

Gene ID	Gene Name	log2FC(DPCs_high vs DPCs_low)	log2FC(DSCs_high vs DSCs_low)
ENSMUSG00000098380	Mir6939	-4.169404915	-7.271711413
ENSMUSG00000112515	AC122235.1	-2.175026169	-6.061404221
ENSMUSG00000065497	Mir410	-2.18589615	-6.047978457
ENSMUSG00000113786	AC099934.7	-5.449729231	-5.556926164
ENSMUSG00000111617	AC133650.2	-3.936125591	-5.555463106
ENSMUSG00000065541	Mir24-2	-4.355034586	-5.554457706
ENSMUSG00000080774	Gm9143	-2.603279489	-5.457933382
ENSMUSG00000074603	Gm10729	-3.080794997	-5.386581053
ENSMUSG00000083505	Gm7541	-4.08456181	-5.313897934
ENSMUSG00000104211	Gm37985	-5.589339044	-5.155425432
ENSMUSG00000091785	Rpl29-ps5	-3.128878462	-5.045221393
ENSMUSG00000032946	Rasgrp2	-7.41875088	-4.99210941
ENSMUSG00000065451	Mir101a	-5.99557862	-4.879989138
ENSMUSG00000109617	Gm7669	-2.505136072	-4.780352855
ENSMUSG00000024598	Fbn2	-8.422004247	-4.635167843
ENSMUSG00000031165	Was	-2.51741927	-4.496132804
ENSMUSG00000030742	Lat	-3.14011356	-4.449740626
ENSMUSG00000030607	Acan	-2.313654519	-4.425161641
ENSMUSG00000081021	Gm11964	-3.866227361	-4.138463181
ENSMUSG00000099088	Gm27920	-2.011972642	-4.062699557
ENSMUSG00000072421	Gm10357	-2.678718712	-4.048324525
ENSMUSG00000081797	Gm8662	-6.113064769	-3.917750657
ENSMUSG00000104806	Gm42566	-2.122404484	-3.888836022
ENSMUSG00000081773	Gm8302	-6.390940305	-3.883546855
ENSMUSG00000077614	Gm25500	-4.97080647	-3.72935241
ENSMUSG00000098516	Mir7651	-2.1351638	-3.709123745
ENSMUSG00000099117	Gm27404	-3.265529623	-3.648460755
ENSMUSG00000020154	Ptprb	-3.319570878	-3.646092191
ENSMUSG00000020953	Coch	-2.024348487	-3.611887758
ENSMUSG00000105796	Gm42845	-2.989927521	-3.432958761
ENSMUSG00000111163	AC125374.2	-2.591866462	-3.422029069
ENSMUSG00000113123	AC166358.2	-3.288673842	-3.36076813
ENSMUSG00000100046	Gm29232	-10.60856765	-3.358269357
ENSMUSG00000053617	Sh3pxd2a	-2.055002806	-3.357806245
ENSMUSG00000004633	Chn2	-2.13260098	-3.265769012
ENSMUSG00000031391	L1cam	-2.506249033	-3.24082471
ENSMUSG00000028373	Astn2	-2.719287926	-3.198378506
ENSMUSG00000091756	Gm3095	-3.244789979	-3.154472164

ENSMUSG00000040086	Tnni3k	-2.529703969	-3.128101559
ENSMUSG00000051457	Spn	-3.35451389	-3.12377682
ENSMUSG00000041482	Piezo2	-9.123242506	-3.083689421
ENSMUSG00000042190	Cmklr1	-3.603758379	-3.019871523
ENSMUSG00000044469	Tnfaip8l1	-2.069888121	-3.018349436
ENSMUSG00000111932	AC124346.1	-2.808033465	-2.974276119
ENSMUSG00000037860	Aim2	-2.413360532	-2.957016571
ENSMUSG00000081642	Gm13532	-3.671702258	-2.949378045
ENSMUSG00000003526	Prodh	-3.311489144	-2.921536494
ENSMUSG00000082828	Gm16106	-2.653534936	-2.912201746
ENSMUSG00000087457	Gm7799	-5.021846193	-2.902579769
ENSMUSG00000081633	Gm8522	-2.709723286	-2.901808324
ENSMUSG00000096049	Gm2075	-3.553539546	-2.901139026
ENSMUSG00000031561	Tenm3	-2.119420916	-2.89619412
ENSMUSG00000105651	Gm43003	-3.010326552	-2.875559604
ENSMUSG00000076119	Mir698	-2.929855391	-2.831882576
ENSMUSG00000048251	Bcl11b	-4.56682564	-2.830976524
ENSMUSG00000023045	Soat2	-5.757859918	-2.791191731
ENSMUSG00000071475	Rpl21-ps5	-2.813989579	-2.761443396
ENSMUSG00000102840	Gm38037	-2.112754219	-2.76133537
ENSMUSG00000053004	Hrh1	-2.737470906	-2.753519604
ENSMUSG00000054293	A630033H20Rik	-5.094199377	-2.753161031
ENSMUSG00000046057	Gm15428	-2.996093228	-2.749080145
ENSMUSG00000089239	Gm22200	-2.098684684	-2.746968857
ENSMUSG00000026955	Sapcd2	-4.598125502	-2.731221439
ENSMUSG00000039252	Lgi2	-3.603700432	-2.711543476
ENSMUSG00000015134	Aldh1a3	-4.715305484	-2.707930236
ENSMUSG00000071151	Gm4799	-2.359111857	-2.707293276
ENSMUSG00000033453	Adamts15	-2.366529181	-2.675238545
ENSMUSG00000044583	Tlr7	-2.743269393	-2.654520101
ENSMUSG00000069913	Anp32-ps	-2.724031168	-2.629507326
ENSMUSG00000048574	Gm5593	-4.217780961	-2.622750619
ENSMUSG00000067321	Gm7931	-2.954504486	-2.583868888
ENSMUSG00000026443	Lrrn2	-2.903822088	-2.557799134
ENSMUSG00000105204	Gm43738	-3.018675189	-2.552247297
ENSMUSG00000044254	Pcsk9	-5.410980798	-2.551754616
ENSMUSG00000057280	Musk	-3.733937498	-2.518322652
ENSMUSG00000053063	Clec12a	-2.183730408	-2.506744079
ENSMUSG00000026715	Serpinc1	-2.365671184	-2.493699256
ENSMUSG00000020788	Atp2a3	-2.087658972	-2.480426194

ENSMUSG00000005338	Cadm3	-2.777678081	-2.461160759
ENSMUSG00000104360	Gm8762	-2.258620195	-2.441644084
ENSMUSG00000106930	Gm6450	-2.503544712	-2.437964703
ENSMUSG00000088856	Gm24727	-2.086189413	-2.433187509
ENSMUSG00000101199	Gm9687	-4.071843303	-2.430919301
ENSMUSG00000026994	Galnt3	-2.342136872	-2.42170968
ENSMUSG00000098149	Gapdh-ps14	-5.293254149	-2.417058885
ENSMUSG00000103891	Gm37941	-2.513091561	-2.402478617
ENSMUSG00000104528	Gm43314	-2.375033022	-2.375341274
ENSMUSG00000082927	Gm5863	-3.8763183	-2.372476582
ENSMUSG00000086644	Gm13470	-3.334232971	-2.360170735
ENSMUSG00000006398	Cdc20	-4.223195299	-2.356438978
ENSMUSG00000031250	Tnmd	-3.031670491	-2.349147023
ENSMUSG00000102908	Gm7558	-2.867949614	-2.344558306
ENSMUSG00000036687	Tmem184a	-5.143486807	-2.341102923
ENSMUSG00000109086	Gm5596	-3.271044388	-2.328263526
ENSMUSG00000065124	Snora65	-2.498414778	-2.304614109
ENSMUSG00000113128	AC139323.1	-3.155349812	-2.29603531
ENSMUSG00000082870	Gm12165	-3.249938045	-2.294144497
ENSMUSG00000036223	Ska1	-3.274450289	-2.282582411
ENSMUSG00000078722	Gm12394	-2.257366123	-2.268908157
ENSMUSG00000028832	Stmn1	-2.488984672	-2.261891666
ENSMUSG00000079259	Trim71	-2.598205618	-2.26092628
ENSMUSG00000043870	Gm5809	-3.944277993	-2.255212207
ENSMUSG00000048416	Mlf1	-3.633968044	-2.236745494
ENSMUSG00000041117	Ccdc8	-5.398800362	-2.225841381
ENSMUSG00000070713	Gm10282	-3.566788304	-2.221514496
ENSMUSG00000101716	Gm12057	-2.081910122	-2.207799194
ENSMUSG00000065259	Snora30	-2.634159227	-2.206542803
ENSMUSG00000074782	4833422C13Rik	-2.278664515	-2.206306366
ENSMUSG00000018983	E2f2	-3.849637325	-2.204739303
ENSMUSG00000051832	E230016K23Rik	-4.453149792	-2.196337173
ENSMUSG00000064620	Gm22303	-2.258483125	-2.170962023
ENSMUSG00000078773	Rad54b	-2.462946917	-2.160091401
ENSMUSG00000099979	Gm5896	-2.808311299	-2.159837887
ENSMUSG00000046410	Kcnk6	-2.498915858	-2.158243765
ENSMUSG00000084845	Tmem240	-2.613493222	-2.156794312
ENSMUSG00000000791	Il12rb1	-4.86379518	-2.155066877
ENSMUSG00000039706	Ldb2	-3.111533605	-2.142594474
ENSMUSG00000024112	Cacna1h	-2.333715603	-2.131376355

ENSMUSG00000024331	Dsc2	-4.403665319	-2.129939104
ENSMUSG00000027276	Jag1	-4.111100255	-2.108877111
ENSMUSG00000089865	Gm44503	-2.864342597	-2.099778318
ENSMUSG00000074476	Spc24	-2.312321912	-2.084915339
ENSMUSG00000106178	Gm42987	-2.813187796	-2.084361943
ENSMUSG00000092887	Snord53	-2.359253394	-2.068325047
ENSMUSG00000038782	1700028J19Rik	-2.87466594	-2.051976154
ENSMUSG00000087120	Gm12279	-2.153818695	-2.043058122
ENSMUSG00000092345	Gm20503	-2.177712698	-2.038353712
ENSMUSG00000000037	Scml2	-4.102354188	-2.025048728
ENSMUSG00000112073	AC131720.3	-2.165538095	-2.023783404
ENSMUSG00000015852	Fcrls	-3.422702775	-2.022129006
ENSMUSG00000027544	Nfatc2	-3.480403767	-2.009717915
ENSMUSG00000061048	Cdh3	-2.516508007	-2.004667072

References:

1.Harries MJ, et al. Lichen planopilaris is characterized by immune privilege collapse of the hair follicle's epithelial stem cell niche. *J Pathol*. 2013;231(2):236-47.
